# Conservation Hotspots of *Quercus castaneifolia* Revealed Through the Integration of Genetic Diversity and Landscape Connectivity

**DOI:** 10.1002/ece3.73393

**Published:** 2026-04-17

**Authors:** Gilda Shahnaseri, Mansoureh Malekian, Markus Müller, Oliver Gailing

**Affiliations:** ^1^ Department of Natural Resources Isfahan University of Technology Isfahan Iran; ^2^ Forest Genetics and Forest Tree Breeding University of Göttingen Göttingen Germany

**Keywords:** conservation genetics, genetic differentiation, genetic diversity, landscape connectivity, *Quercus*

## Abstract

*Quercus castaneifolia* C.A. Mey., a dominant and ecologically important oak species in the Hyrcanian forests of northern Iran, is experiencing rapid decline due to climate change, anthropogenic pressures, and severe habitat fragmentation. This study aims to delineate genetic diversity patterns and identify key conservation areas for *Q. castaneifolia* across its entire range within Iran. We integrated population genetic analyses with landscape connectivity modeling to identify regions of high evolutionary and ecological importance. Using 14 polymorphic nuclear microsatellite (nSSR) and four chloroplast microsatellite (cpSSR) loci, we assessed 235 individuals across the species' geographical distribution. Nuclear markers revealed high genetic diversity (*H*_e_ = 0.54) and weak differentiation (*F*_ST_ = 0.019), suggesting extensive pollen flow, while cpSSRs showed strong spatial structure and restricted seed dispersal. Seven distinct chloroplast haplotypes were identified, with the Talesh–Mardab hydro‐region exhibiting the highest haplotype diversity and harboring four private haplotypes. Habitat connectivity analysis using UNICOR revealed two main core areas located in the western (Talesh–Mardab) and eastern (Qarasu–Gorgan) Hyrcanian regions, connected by a central corridor through Lahijan–Nur and Haraz–Naka. These spatial patterns closely overlapped with areas of haplotype diversity, highlighting them as conservation hotspots. Our nuclear SSR results are consistent with previous findings showing low differentiation and extensive pollen flow, whereas cpDNA markers provided additional insights into seed dispersal and spatially structured haplotype variation. Our integrated approach provides a valuable framework for identifying core populations and genetic diversity hotspots, as well as for preserving gene flow pathways essential to the species' long‐term persistence and its dynamic gene conservation.

## Graphical abstract

This study integrates the genetic characterization of chestnut‐leaved oak across its distribution range in the Hyrcanian forests with landscape connectivity modeling to identify regions of high evolutionary and ecological importance. Two main core areas, located in the western and eastern parts of the Hyrcanian forests, were identified and connected by a central corridor. The spatial patterns closely overlapped with areas of elevated allelic richness and haplotype diversity, highlighting these regions as conservation hotspots.

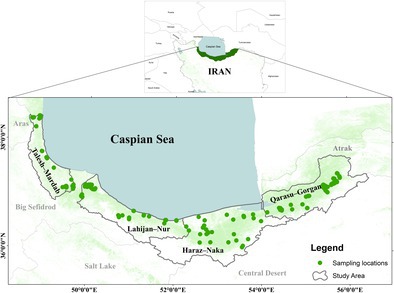

## 1 Introduction

The Hyrcanian forest, also known as the Caspian forest, is a unique and ancient temperate ecosystem extending along the southern coast of the Caspian Sea in Azerbaijan and Iran. As relicts of the Tertiary period, these forests harbor a high proportion of endemic and phylogenetically ancient broad‐leaved tree species, and have functioned as long‐term refugia during Quaternary climatic oscillations (Akhani et al. 2010; Sagheb‐Talebi et al. 2014). Its ecological uniqueness and outstanding biodiversity values led to its designation as a UNESCO World Heritage Site (UNESCO 2019). Among its keystone tree species, the chestnut‐leaved oak (*Quercus castaneifolia* C.A. Mey.) plays a central role in shaping forest structure, regulating microclimatic conditions, and supporting a wide range of forest‐dependent organisms (Vakili et al. 2021). This species is a forest‐forming oak that contributes substantially to canopy architecture, microclimatic regulation, and stand‐level heterogeneity across a wide elevational gradient in the Alborz Mountains (Sagheb‐Talebi et al. 2014). *Q. castaneifolia* commonly occurs in the structurally and genetically diverse Eurasian relict Hyrcanian mixed deciduous forests of northern Iran. It coexists both in mixed stands and in association with other ecologically important tree species such as *Parrotia persica* , *Acer velutinum*, *Gleditsia caspica*, *Alnus subcordata*, and *Pterocarya fraxinifolia*, contributing to the high biodiversity and complex forest composition of these relict ecosystems. As a dominant canopy species within these assemblages, changes in the abundance or spatial continuity of *Q. castaneifolia* are expected to have cascading effects on forest composition, regeneration dynamics, and biodiversity maintenance (Rix and Kirkham 2009; Le Hardÿ de Beaulieu and Lamant 2010; Coombes 2014; Vatanparast et al. 2024). Although this species occurs across a broad elevational gradient, it occupies only about 6.5% of the Hyrcanian forest area and faces multiple anthropogenic threats, including illegal thinning, weak regeneration in lowland sites, and habitat fragmentation (Shahnaseri et al. 2023). Human activities such as road expansion, logging, and land‐use conversion further exacerbate forest degradation, while changing climate regimes intensify these impacts through rising temperatures, altered precipitation patterns, and increased frequency of extreme events (Taleshi et al. 2019; Shahnaseri et al. 2023). Together, these drivers threaten population viability, recruitment dynamics, and forest regeneration, emphasizing the need for science‐based conservation strategies.

*Quercus castaneifolia* thrives across a wide range of soil and climatic conditions, with annual precipitation ranging from 150 to 2000 mm and mean annual temperatures between 10°C and 18°C (Asadi et al. 2025). It forms extensive stands along nearly 800 km of the northern slopes of the Alborz Mountains, acting as an ecological backbone of the Hyrcanian temperate deciduous forest (Sagheb‐Talebi et al. 2014). Nevertheless, ecological adaptability alone may not be sufficient to buffer against genetic erosion and population decline. Numerous studies on oaks and other long‐lived forest trees have shown that demographic bottlenecks and habitat fragmentation can reduce evolutionary potential and adaptive capacity, ultimately limiting the ability of populations to respond to rapid environmental change (Fady et al. 2016; Kumar et al. 2024). These insights have underscored the need for conservation strategies that move beyond population‐level summaries and explicitly consider spatial context.

From a taxonomic and evolutionary perspective, *Q. castaneifolia* belongs to *Quercus* section *Cerris*, a Eurasian oak lineage characterized by relatively recent diversification and partially permeable species boundaries, as shown by phylogenomic analyses of the genus *Quercus* (Hipp et al. 2020). Species within this section are well known for frequent interspecific hybridization and introgression, which can complicate the interpretation of population genetic patterns when closely related taxa occur in sympatry (Hipp et al. 2020; Curtu et al. 2007). Phylogenetically, *Q. castaneifolia* is closely related to other *Cerris* taxa such as *Q. cerris* , *Q. libani*, and *Q. ithaburensis* , for which hybridization has been documented, particularly in areas where distribution ranges overlap (Curtu et al. 2007; Hipp et al. 2020). Nevertheless, the geographical distribution of *Q. castaneifolia* is largely restricted to the Hyrcanian forests along the southern Caspian Sea and adjacent parts of the Caucasus, whereas most other *Cerris* species occur mainly in Anatolia, the eastern Mediterranean, or the Zagros region, resulting in limited spatial overlap (Sagheb‐Talebi et al. 2014; Hipp et al. 2020). This pronounced spatial segregation suggests that the probability of ongoing hybridization or introgression with other *Cerris* taxa within the Hyrcanian forests is low, and that the genetic patterns observed in the present study predominantly reflect intraspecific evolutionary and demographic processes. However, limited present‐day spatial overlap does not entirely rule out historical contacts or deeper evolutionary relationships within section *Cerris*. Phylogenomic evidence indicates that several *Cerris* species share plastid signatures and complex lineage affinities (Denk et al. 2023), suggesting that past hybridization, plastid capture, or retention of ancestral polymorphism may have contributed to the broader evolutionary context of this clade. Population genetics provides a fundamental framework for biodiversity conservation by quantifying genetic diversity, detecting population substructure, and identifying potential risks associated with demographic changes or inbreeding (Frankham et al. 2018). However, genetic patterns alone often provide limited insight into the spatial processes and landscape features that shape gene flow across heterogeneous environments. Over the past two decades, advances in spatially explicit analyses have increasingly bridged this gap by integrating genetic data with geographic and environmental information (Degen and Scholz 1998). In this context, mapping spatial variation in genetic indices such as allelic richness or haplotype diversity, together with resistance‐based connectivity modeling, has become a widely adopted and effective approach for identifying conservation‐relevant units, genetic diversity hotspots, and functional corridors across complex landscapes (Hanotte et al. 2002; Hoffmann et al. 2003; Van Zonneveld et al. 2012). While these approaches are no longer novel, they remain highly relevant for applied conservation, particularly for long‐lived forest trees, where maintaining both genetic diversity and landscape connectivity is essential for long‐term persistence under ongoing environmental change.

In this context, molecular markers, particularly nuclear and chloroplast microsatellites, are powerful tools for assessing genetic variation, population structure, and connectivity, thereby providing essential information for the effective management of forest tree species (Finkeldey and Hattemer 2007; Porth and El‐Kassaby 2014). Nuclear SSRs (nSSRs) reflect biparental inheritance and current pollen‐mediated gene flow, whereas chloroplast SSRs (cpSSRs), which are maternally inherited in most angiosperms, trace historical seed‐mediated dispersal and phylogeographic structure (Hampe and Petit 2005; Pazouki et al. 2016). Dual‐marker approaches combining nSSRs and cpSSRs have proven particularly informative in *Quercus* species, elucidating how postglacial recolonization, seed dispersal limitation, and historical landscape dynamics shape contemporary genetic patterns (Hampe and Petit 2005; Crăciunesc et al. 2016; Simeone et al. 2018).

Consequently, integrating population genetic analyses with landscape connectivity modeling provides an effective framework for exploring how spatial heterogeneity and habitat permeability influence gene flow and the spatial distribution of genetic diversity. This approach is particularly relevant for forest trees experiencing habitat fragmentation and climatic change, as it facilitates the identification of genetic diversity hotspots and functionally connected corridors for conservation planning (Sork et al. 2013; McKinney et al. 2017; Feng and Du 2022).

Recent genomic research on *Q. castaneifolia* using genome‐wide SNP markers (RADseq) revealed low but significant genetic differentiation between western and central–eastern populations, consistent with extensive pollen‐mediated gene flow across the Hyrcanian region (Vatanparast et al. 2024). However, spatially explicit assessments that integrate nuclear and chloroplast markers with landscape connectivity analyses are still lacking for this species. Such analyses are essential for identifying genetically important populations, landscape areas preserving maternally inherited (chloroplast) genetic structure resulting from limited seed dispersal and habitat stability, and connectivity corridors that may be particularly vulnerable to ongoing habitat fragmentation and forest degradation documented in recent decades. In this study, we employed an integrative population genetic and landscape connectivity framework to investigate genetic diversity, structure, and connectivity in *Q. castaneifolia* across its entire distribution range in Iran. Specifically, we aimed to (1) evaluate the transferability and polymorphism of nuclear and chloroplast SSR markers developed from related oak species to *Q. castaneifolia*; using the polymorphic and reliable loci identified, (2) assess the level and distribution of genetic diversity in *Q. castaneifolia* across the Hyrcanian forests; (3) determine population structure and gene flow patterns, distinguishing between predominantly pollen‐mediated (nSSR‐based) and seed‐mediated (cpSSR‐based) gene flow; and (4) identify genetic diversity hotspots, populations with private haplotypes, and potential connectivity corridors among core habitats for conservation prioritization. Additionally, we sought to examine whether patterns revealed by nSSR and cpSSR markers corroborate the genome‐wide findings of Vatanparast et al. (2024), thereby contributing complementary and spatially explicit evidence to strengthen conservation strategies for this keystone forest tree.

## 2 Material and Methods

### 2.1 Tree Sampling and Study Area

**FIGURE 1:**
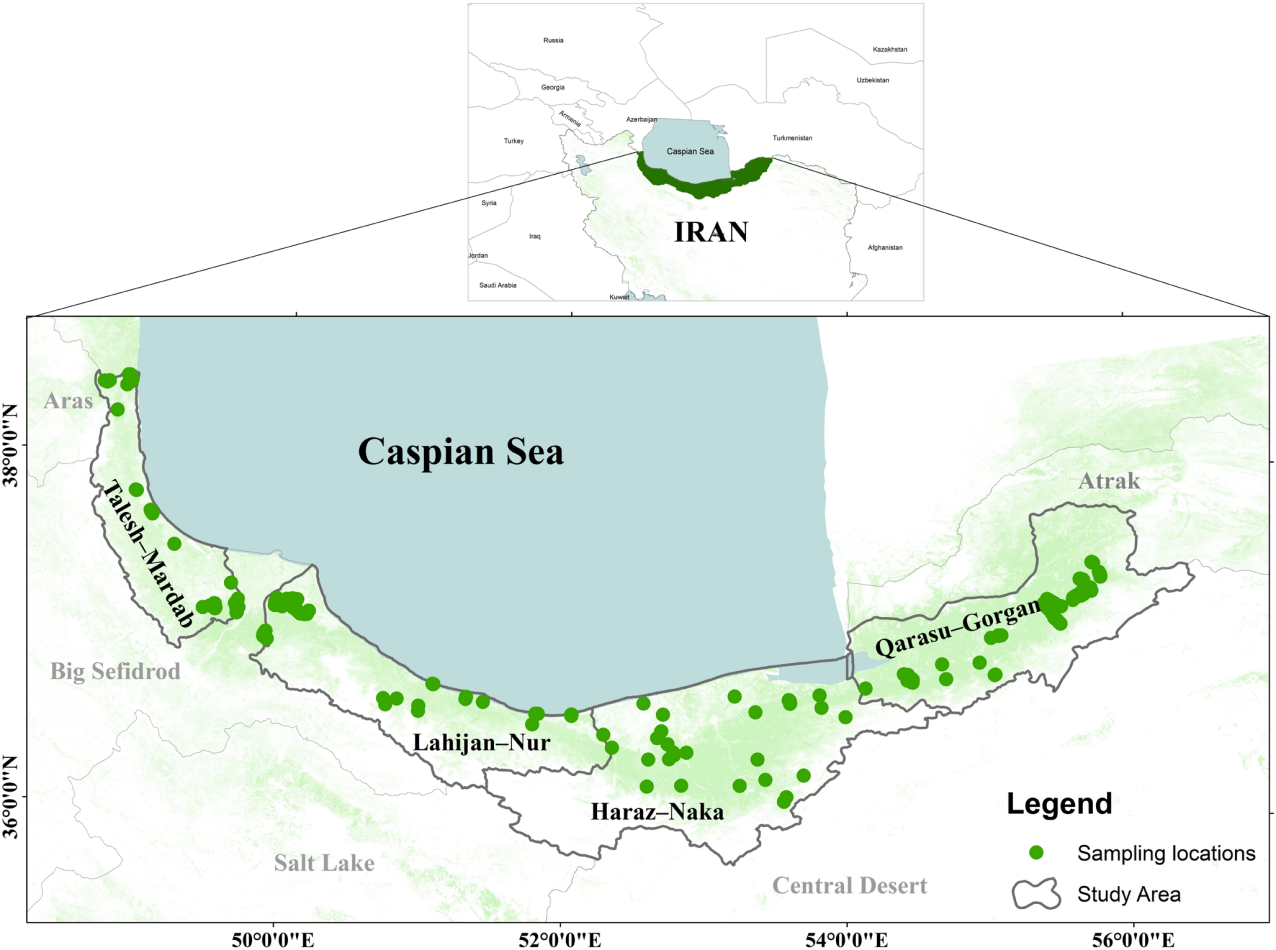
Sampling plots of *Q. castanifolia* across the four hydro‐regions (Talesh–Mardab, Lahijan–Nur, Haraz–Neka, and Qarasou–Gorgan) in Iran (a total of 45 plots).

This region extends along the northern slopes of the Alborz Mountain range and the southern coast of the Caspian Sea, covering latitudes 35°48′ N to 37°55′ N and longitudes 48°31′ E to 56°10′ E, encompassing a total area of approximately 1.85 million hectares (Figure 1). A systematic‐random sampling approach was used to ensure comprehensive coverage of the study area. The region was divided into a grid with cells measuring 20 × 20 km, and one sampling site was randomly selected within each cell based on geographic coordinates generated by GIS software. This approach was chosen to ensure diverse representation of different environmental and ecological conditions within the region. However, only grid cells containing suitable forest habitat and populations of *Q. castaneifolia* were sampled, while cells dominated by non‐forest land uses (e.g., agriculture or urban areas) were excluded. This resulted in an apparently uneven spatial distribution of sampling sites (Figure 1). Existing forest inventory data provided by the Iranian Forests, Range, and Hydro‐Region Management Organization were consulted as auxiliary guidance to identify grid cells harboring the target species. A total of 235 oak trees were sampled from approximately 45 sampling plots across the Hyrcanian forests, which extend from the western to the eastern regions along the entire southern shoreline of the Caspian Sea. From each plot, at least five trees were randomly selected to provide adequate representation of the genetic diversity within the area. The four major hydro‐regions (Talesh–Mardab, Lahijan–Nur, Haraz–Neka, and Qarasou–Gorgan) were considered as hydro‐regions for genetic analyses, as they represent distinct hydrological and environmental conditions potentially influencing the genetic structure of *Q. castaneifolia*. These habitats range from areas receiving less than 600 mm of annual mean precipitation and an annual mean temperature of 10°C in the east, to regions where precipitation and air temperature exceed annual means of 2000 mm and 16°C, respectively (Hamidi et al. 2023, Figure S1). This sampling design ensured that the entire climatic and elevational gradient of the Hyrcanian forests was adequately represented, minimizing spatial autocorrelation between sites.

The field survey was conducted between April and June 2023, coinciding with the period of maximum leaf development to ensure high‐quality tissue for DNA extraction. At each site, one mature and healthy tree was sampled to avoid collecting from damaged or diseased individuals. To prevent cross‐contamination among samples, leaves from each individual were placed in separate plastic bags containing 30 g of silica gel and labeled properly until DNA extraction and genotyping.

### 2.2 DNA Extraction

Genomic DNA was extracted from leaf tissue using the DNeasy 96 Plant Kit (Qiagen, Hilden, Germany) in accordance with the manufacturer's protocol. DNA quality was assessed by agarose gel electrophoresis, and samples were diluted for PCR amplification.

#### 2.2.1 Nuclear Microsatellites

Thirty‐three nuclear SSR markers were used for genotyping, including nine genomic SSRs (gSSRs) and 24 EST‐SSRs (Table S1). The gSSRs were originally developed for *Quercus rubra* (Sullivan et al. 2013) and other *Quercus* species (Aldrich et al. 2002). Most EST‐SSRs were developed for the closely related species *Q. robur* and *Q. petraea* (Durand et al. 2010), while additional EST‐SSRs were derived from a *Q. rubra* EST library (Müller and Gailing 2018; Burger et al. 2018). SSR motifs were identified within assembled oak unigene EST sequences using minimum repeat thresholds of five, four, three, three, and three repeats for di‐, tri‐, tetra‐, penta‐, and hexa‐nucleotide motifs, respectively (Durand et al. 2010).

All nuclear SSR loci were initially screened in eight individuals from different parts of the species' distribution to assess amplification success and polymorphism (Table S1). Four multiplex sets were used for nSSR loci (see Table S1 for locus composition). Genomic DNA from each sample was amplified for nSSR loci using four multiplex sets. Each 15 μL PCR reaction contained 1.5 μL reaction buffer (0.8 M Tris–HCl, 0.2 M (NH_4_)_2_SO_4_), 1.5 μL MgCl_2_ (25 mM), 1 μL dNTPs (2.5 mM each), 0.2 μL HOTFIREPol Taq polymerase (Solis BioDyne, Estonia; 5 units/μL), and 1 μL genomic DNA (~0.6 ng/μL). Forward and reverse primers (each 5 picomole/μL) were added at volumes optimized for each multiplex, and the remaining volume was adjusted with nuclease‐free water. Multiplex 1 included quru‐CA‐2P24 and quru‐CA‐3A05 (0.5 μL each primer pair), multiplex 2 contained FIR028, FIR035 (0.5 μL each), VIT107 (0.7 μL), and quru‐GA‐1C06 (0.4 μL), multiplex 3 included FS_C2660 and FS_C8183 (0.2 μL each), PIE223, QrC0057, and QrC0332 (0.25 μL each), and multiplex 4 contained GOT040 and PIE040 (0.5 μL each) and PIE125 (0.4 μL).

PCR amplifications for nSSR loci were performed on a Biometra Thermal Cycler (MJ Research PTC 200, Analytik Jena, Germany) using a touchdown program adapted for *Quercus* microsatellites (Burger et al. 2018). The cycling profile consisted of an initial denaturation at 95°C for 15 min, followed by 10 cycles at 94°C for 1 min, 1 min at 60°C (decreasing 1°C per cycle) and 1 min at 72°C, followed by 25 cycles at 94°C for 1 min, annealing at 50°C for 1 min and elongation at 72°C for 1 min, and a final extension at 72°C for 20 min. Amplification products were separated on an ABI 3130xl Genetic Analyzer (Applied Biosystems, Foster City, USA), and allele sizes were determined using GeneMapper version 4.1 software. Forward primers were fluorescently labeled to allow accurate allele identification.

#### 2.2.2 Chloroplast Microsatellite (cpSSRs) Markers

An initial screening of 42 chloroplast SSR markers, including universal primers (ccmp1–ccmp10; Weising and Gardner 1999), *Quercus*‐specific primers (μdt1–μdt6, μcd1–μcd8, μkk1–μkk4; Deguilloux et al. 2003), and *Q. rubra* ‐specific primers (QRcp01–QRcp041; Götz and Gailing 2022) was conducted on eight individuals representing different parts of the species' distribution (Table S2). Amplification success and polymorphism were evaluated across these test samples. Based on these criteria, four cpSSR markers (QR28, QR29, ccmp6, and μcd4) consistently amplified and showed polymorphism and were thus selected for genotyping the entire study population. These markers were subsequently used to distinguish chloroplast haplotypes across the sampled populations.

PCR amplifications were performed in a Biometra Thermal Cycler (MJ Research PTC 200, Analytik Jena, Germany). For the *Q. rubra* ‐specific markers QR28 and QR29, a touchdown program was applied: initial denaturation at 95°C for 15 min, followed by 10 cycles at 94°C for 1 min, 1 min at 60°C (decreasing 1°C per cycle), and 1 min at 72°C, then 25 cycles at 94°C for 1 min, 50°C annealing for 1 min, and 72°C elongation for 1 min, with a final extension at 72°C for 20 min. For the universal ccmp6 marker and the *Quercus*‐specific marker μcd4, PCR consisted of an initial denaturation at 95°C for 15 min, followed by 30 cycles at 94°C for 1 min, annealing at 50°C for 1 min, elongation at 72°C for 1 min, and a final extension at 72°C for 20 min. PCR products were separated using an ABI 3130xl Genetic Analyzer, and allele sizes were determined with GeneMapper version 4.1 software (Applied Biosystems, Foster City, USA). Forward primers were fluorescently labeled (6‐FAM or HEX; QR28 and QR29) via M13 tail (Schuelke 2000; Kubisiak et al. 2013) for accurate allele identification.

### 2.3 Genetic and Statistical Analysis

To detect the presence of null alleles, we used MICRO‐CHECKER v.2.2.3 (Van Oosterhout et al. 2004). Genetic diversity estimates, including the number of alleles per locus (*N*_a_), effective number of alleles (*N*_ae_), allelic richness standardized to a sample of 74 gene copies (A_R_), observed and expected heterozygosity (*H*_o_ and *H*_e_), and the inbreeding coefficient (*F*_IS_), were calculated for the four hydro‐regions using SPAGeDi 1.5d (Hardy and Vekemans 2002) across all loci, as well as for each SSR marker individually.

Differences in genetic diversity parameters, such as observed and expected heterozygosity, among the four hydro‐regions were assessed using a Kruskal–Wallis test with multiple comparisons, implemented through the R package pgirmess v.1.6.7 (Giraudoux 2021). Additionally, relationships between heterozygosity and environmental factors (soil acidity, precipitation, and temperature) were examined using Pearson's correlation coefficients. To visualize these correlations, scatter smooth plots were created using the R package stats v.4.0.5 (R Core Team 2020). Mantel tests were used to analyze isolation by distance (IBD) and isolation by adaptation (IBA). For IBD, the relationship between genetic distance (Nei's genetic distances) and geographic distance (Euclidean distance) was assessed. For IBA, the relationship between genetic differentiation and environmental dissimilarity was analyzed. The distance matrices were calculated in GenAlEx.

Principal Coordinate Analysis (PCoA) and a Bayesian clustering algorithm via STRUCTURE v2.3.4 (Pritchard et al. 2000) were employed to examine genetic relationships and structure among adult individuals across the four hydro‐regions. PCoA was executed using GenAlEx v.6.5 (Peakall and Smouse 2006) based on pairwise genetic distances between individual samples calculated using codominant genotypic data. For the STRUCTURE analysis, we implemented the admixture model with correlated allele frequencies, testing *K* values ranging from 1 to 5 with 10 replicates for each *K*. Each run consisted of a burn‐in period of 100,000 iterations followed by 1,000,000 Markov Chain Monte Carlo (MCMC) iterations. The most likely value of *K* was determined using the ΔK method (Evanno et al. 2005) via the web‐based STRUCTURE SELECTOR (Li and Liu 2018). The optimal *K* was visualized through bar plots generated by the CLUMPAK pipeline (Kopelman et al. 2015), which clusters similar STRUCTURE runs to ensure consistency.

Allele size analysis was conducted using GeneMapper version 4.1 (Applied Biosystems, Foster City, CA, USA). Haplotype frequencies and distributions based on all the polymorphic chloroplast markers were determined using Haplotype Analysis version 1.05 software (Eliades and Eliades 2009; Georg‐August University, Goettingen, Germany). The haplotype network, which visualizes relationships among haplotypes, was generated using Network 5.0.1.1 (Bandelt et al. 1999). Initially, a “rdf” file was created in GenAlEx 6.5, reformatted, and saved in Network 5.0.1.1 as an “ych” file, which served as the input for generating the haplotype network using the median joining method. The resulting output file was saved for visualization purposes.

#### 2.3.1 Habitat Connectivity and Identification of Conservation Hotspots

Habitat suitability for *Q. castaneifolia* was modeled across the Hyrcanian forests using the maximum entropy algorithm (MaxEnt v3.4.4; Phillips et al. 2006). Initially, 315 presence records were collected during field surveys in April and June 2023. To assess potential spatial clustering, Moran's *I* (*I* = 0.23, *p* < 0.05) was calculated, revealing significant spatial autocorrelation among some points. To reduce sampling bias, spatial filtering was applied by retaining only one individual per 1 km^2^ grid cell, resulting in 235 occurrence records used for modeling. These presence records were combined with climatic (mean annual temperature, annual precipitation), topographic (elevation, slope), and anthropogenic (distance to roads, land use intensity) predictors. All environmental layers were standardized to a 1 km spatial resolution, and highly correlated variables (|*r*| > 0.7) were excluded based on Pearson's correlation analysis.

Model performance was assessed using the Area Under the Curve (AUC) and True Skill Statistic (TSS), and the final suitability map was produced as an ensemble of 10 replicated MaxEnt runs, representing the mean probability of suitable habitat between 0 (unsuitable) and 1 (highly suitable).

To estimate landscape resistance, we converted the habitat suitability maps (ensemble model) to resistance maps using a negative exponential function (Wan et al. 2019) (Equation 1):

(1) R=1000^−1×HS

where *R* represents the cost resistance value assigned to each pixel and HS represents the predicted habitat suitability derived from the suitability models described above (Wan et al. 2019).

Resistance values were then rescaled between 1 and 100 by linear interpolation, such that minimum resistance (*R*_min_ = 1) corresponded to HS = 1 (high suitability) and maximum resistance (*R*_max_ = 100) corresponded to HS = 0 (unsuitable habitat) (Wan et al. 2019).

This transformation assumes that areas with higher environmental suitability impose lower resistance to movement and thus greater permeability for dispersal.

The resulting resistance surface was generated at 1 km spatial resolution and clipped to the Hyrcanian forest boundary to ensure consistency with the genetic sampling extent.

We employed the Universal Corridor Network Simulator (UNICOR v2.0; Landguth et al. 2012) to produce two complementary connectivity predictions: (1) Resistant kernels (Compton et al. 2007), and (2) Factorial least‐cost paths (Cushman et al. 2009).

The factorial least‐cost path analysis in UNICOR applies Dijkstra's algorithm to compute the shortest paths between all mapped occurrence locations of *Q. castaneifolia*, summing all pairwise least‐cost paths to create a corridor density map (Landguth et al. 2012).

The resistant kernel algorithm calculates a cumulative resistance cost‐weighted dispersal kernel around each source point up to a user‐defined dispersal threshold (here, 30 km), providing an incidence function of potential movement rate through each pixel as a function of source density, dispersal ability, and landscape resistance (Compton et al. 2007).

Both outputs were standardized and visualized in ArcGIS Pro 3.2. Cells above the 95th percentile of connectivity probability were defined as core habitats, while continuous high‐density connections among them were delineated as corridors. These connectivity maps were subsequently compared with allelic richness and chloroplast haplotype diversity patterns to identify genetic–ecological conservation hotspots.

## 3 Results

### 3.1 Genetic Variation Across Populations Using nSSRs

A total of 33 nuclear SSR markers were tested for amplification in *Q. castaneifolia*. This set comprised nine gSSRs and 17 EST‐SSRs originally developed for *Q. robur* and *Q. petraea* , plus seven EST‐SSRs developed for North American red oak ( *Q. rubra* ) (Table S1). Of these, 60.6% (20 markers) successfully amplified, with 42.4% (14 markers) being polymorphic. MICRO‐CHECKER identified a low frequency of null alleles at only one locus (FIR028) (Table S3). Genetic diversity levels were comparable across the four hydro‐regions (Table 1). Additionally, the coefficients of inbreeding (*F*_IS_) across all markers were not significantly different from zero in any population (Table 1). The genetic parameters for each locus are summarized in Table S4. The mean expected heterozygosity (*H*_e_) per locus ranged from 0.11 (FS‐C8183) to 0.89 (FIR028), while the mean observed heterozygosity (*H*_o_) ranged from 0.11 (FS_C8183) to 0.69 (PIE223). The markers exhibited a broad range of *F*_IS_ values, from −0.02 to 0.28, with two markers (quru‐GA‐1C06 and FIR028) showing negative *F*_IS_ values, indicating an excess of heterozygotes (Table S4). The Kruskal–Wallis test indicated that there were no significant differences in observed and expected heterozygosity among the four hydro‐regions (*p* > 0.05).

**TABLE 1:** Genetic variation over nSSR markers without null alleles for the *Q. castaneifolia* hydro‐regions of the Hyrcanian forest.

Hydro‐regions	*N*	*N*_a_	*N*_ae_	A_R_	*H*_e_	*H*_o_	*F*_IS_	*p* value for *F*_IS_
Talesh–Mardab	41.63	5.63	2.83	5.41	0.54	0.52	−0.01	0.63
Lahijan–Nur	64.54	5.63	2.96	5.18	0.54	0.48	0.06	0.04
Haraz–Naka	38.63	5.00	3.00	4.95	0.54	0.47	0.05	0.02
Qarasu–Gorgan	88.72	5.90	2.93	5.05	0.55	0.51	0.09	0.02
Mean	58.38	5.54	2.93	5.22	0.54	0.50	0.05	0.00

Abbreviations: A_R_ (*k* = 74), allelic richness of a standardized sample of 74 gene copies; *F*_IS_, inbreeding coefficient; *H*_e_, expected heterozygosity; *H*_o_, observed heterozygosity; *N*, number of samples; *N*_a_, number of alleles; *N*_ae_, effective number of alleles.

**FIGURE 2:**
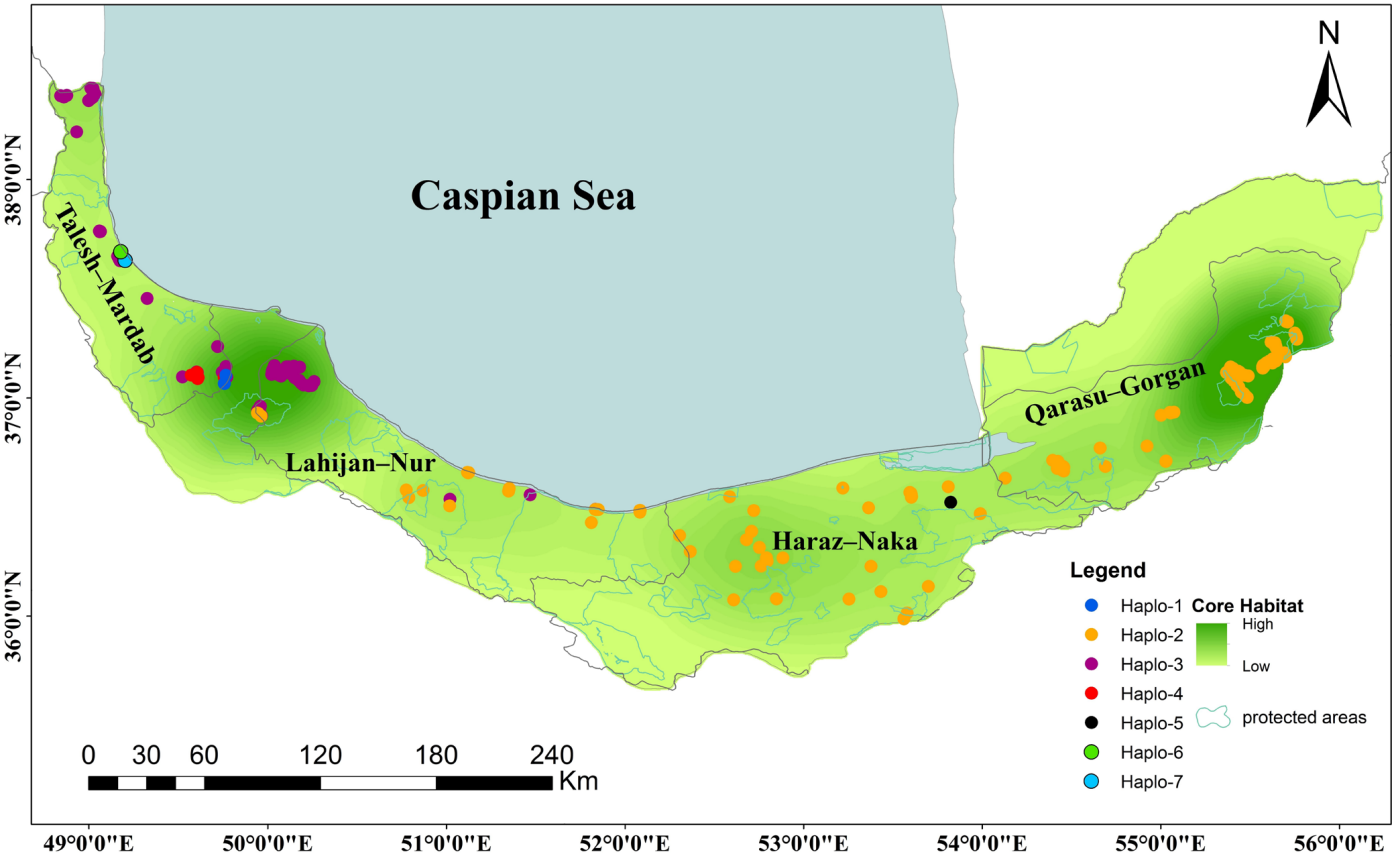
Distribution of cpDNA haplotypes among *Q. castanifolia* populations, along with the location of Iranian protected areas, overlaid on the core habitat probability predicted by UNICOR.

The PCoA analysis based on genetic distances among all individuals from the four hydrological regions showed no clear clustering by four hydro‐region or environmental factors, suggesting the absence of detectable genetic structure related to these variables (Figure S2). Similarly, STRUCTURE analysis also failed to identify distinct genetic clusters, with the highest probability observed for *K* = 1, further indicating a lack of significant population structure (Figure 2 and Figure S4).

Mantel tests assessing IBD and IBA revealed no significant correlation between genetic distance and geographic distance (*r* = 0.02, *p* = 0.41) nor between genetic distance and environmental distance (*r* = −0.05, *p* = 0.72). In the case of *Q. castaneifolia*, neither geographic barriers nor the measured environmental variables (precipitation, temperature, and soil pH) significantly influenced the genetic structure of *Q. castaneifolia* populations.

### 3.2 Genetic Variation Across Populations Using cpSSRs

For cpSSR markers, 64.3% (27 out of 42 tested primers) successfully amplified, but only 9.5% (4 markers) were polymorphic (Table S2).

A total of seven distinct chloroplast haplotypes were identified across the Hyrcanian forest (Figure 2). Of these, five haplotypes were found in Talesh–Mardab, with four being unique to this region. In Lahijan–Nur, two haplotypes were detected, with no private haplotypes. Haraz–Naka exhibited two haplotypes, including one private haplotype. In contrast, Qarasu–Gorgan exhibited only a single haplotype, with no private haplotypes (Figure 2).

The Talesh–Mardab hydro‐region exhibited the highest haplotype diversity, with an effective number of haplotypes *N*_e_ of 1.75 and haplotypic richness R_h_ of 3.800 (Table 2). These values suggest that Talesh–Mardab may serve as a key area of haplotype diversity for *Q. castanifolia* in the Hyrcanian forest. In comparison, the Qarasu–Gorgan region showed no haplotypic variation, while Lahijan–Nur and Haraz–Naka displayed intermediate diversity levels (*N*_e_ = 1.81 and 1.11, R_h_ = 2.00 and 2.00, respectively) (Table 2; Table S5). Although Lahijan–Nur showed slightly higher *N*_e_ values, the absence of private haplotypes indicates shared rather than unique chloroplast diversity compared to Talesh–Mardab. The presence of four private haplotypes in Talesh–Mardab (Figure 2, Table 2) highlights the unique genetic diversity of this region, which is absent in other populations. Similarly, Haraz–Naka harbored one private haplotype, underscoring the localized genetic diversity in these areas.

**TABLE 2:** Chloroplast haplotype diversity within the *Q. castanifolia* populations of the Hyrcanian forest.

Population	*N*	A	P	*N*_e_	R_h_	*H*_e_	D^2^_sh
Talesh–Mardab	42	5	4.00	1.75	3.80	0.43	0.55
Lahijan–Nur	65	2	0	1.81	2.00	0.45	1.81
Haraz–Naka	38	3	1	1.11	2.00	0.10	0.25
Qarasu–Gorgan	84	1	0	1.00	0.00	0.00	0.00
Mean	57.25	2.75	1.25	1.41	1.70	0.24	0.65

Abbreviations: A, number of haplotypes detected; D^2^_sh, mean genetic distance between individuals; *H*_e_, Nei's gene diversity; *N*, number of samples; *N*_e_, effective number of haplotypes; P, number of private haplotypes; R_h_, haplotypic richness.

**FIGURE 3:**
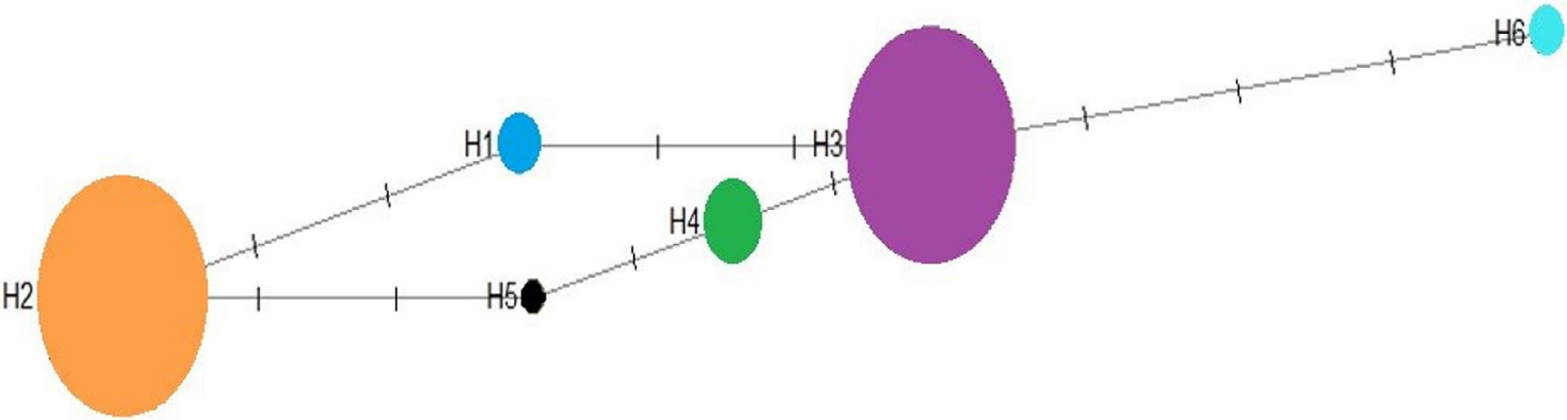
Haplotype network of chloroplast haplotypes in *Q. castanifolia* populations (H1–H7). Circle size represents the frequency of each haplotype. Lines connecting haplotypes are indicated by vertical bars representing hypothetical mutations. Although vertical line distances are uniform, some lines were condensed for visualization clarity.

The haplotype network (Figure 3) reveals distinct genetic clusters, with private haplotypes in Talesh–Mardab and Haraz–Naka, suggesting potential geographic isolation of these populations. The distribution of haplotypes suggests limited seed‐mediated gene flow, particularly between the westernmost population (Talesh–Mardab) and the easternmost population (Qarasu–Gorgan), likely due to geographic isolation.

### 3.3 Habitat Connectivity and Identification of Conservation Hotspots

**FIGURE 4:**
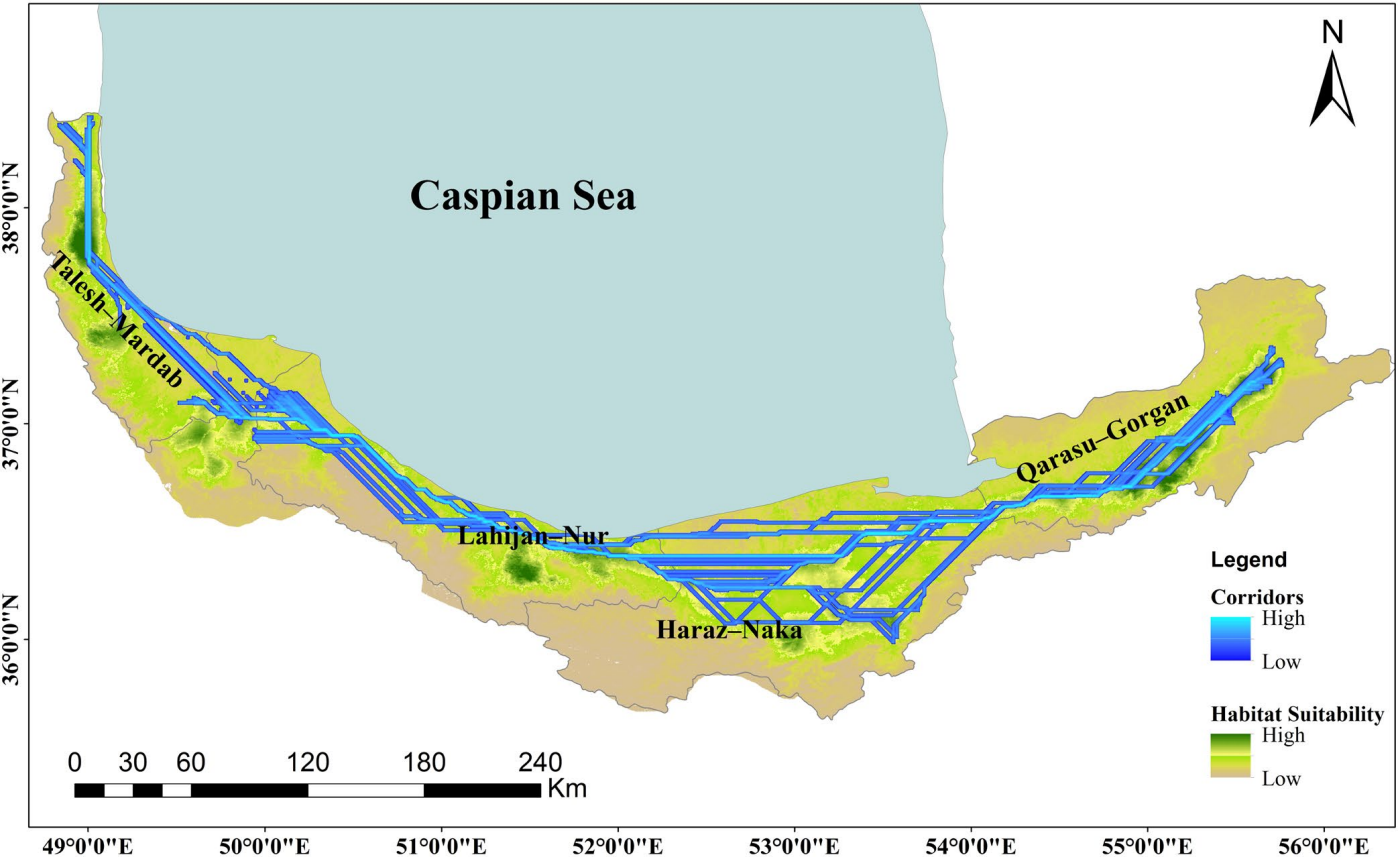
Connectivity corridors (blue–cyan) and habitat suitability (green gradient) of *Q. castaneifolia* across the Hyrcanian forests, linking major suitable areas identified by resistance‐based connectivity modeling.

To assess whether landscape connectivity is consistent with spatial genetic patterns, habitat suitability and resistance surfaces were integrated into a connectivity framework using UNICOR. The resulting maps identified two main core areas in the western (Talesh–Mardab) and eastern (Qarasu–Gorgan) Hyrcanian regions, connected by a continuous but narrow corridor passing through the Lahijan–Nur and Haraz–Naka areas (Figures 2 and 4). Although no population structure was detected based on nuclear SSR markers (*K* = 1), corridor density reached its maximum in the central Hyrcanian range. This region spatially coincides with the transition zone between eastern and western lineages identified by maternally inherited cpSSR markers and by genome‐wide RADseq analyses (*K* = 2; Vatanparast et al. 2024). The persistence of this east–west differentiation despite high corridor density suggests that landscape connectivity primarily reflects spatial permeability rather than sufficient effective seed‐mediated gene flow to fully homogenize chloroplast genetic variation.

Overall, the UNICOR results delineate two major conservation hotspots (Talesh–Mardab and Qarasu–Gorgan) connected by a central corridor. These findings highlight the spatial overlap between areas of high habitat stability and genetic diversity, emphasizing their conservation priority for maintaining landscape‐level genetic connectivity in *Q. castaneifolia*.

## 4 Discussion

This study demonstrates the successful transfer and polymorphism of both nuclear and chloroplast markers in *Q. castaneifolia* originally developed for *Q. rubra* . Combining these genetic data with resistance‐based landscape connectivity modeling using the UNICOR framework revealed that areas of high genetic diversity coincide with ecologically stable core habitats throughout the Hyrcanian forests.

The concentration of distinct haplotypes and core habitats identified in this study supports the role of the Hyrcanian forests as an important refugial area for oak species during past climatic fluctuations. Such refugia are expected to harbor elevated genetic diversity and contribute to the long‐term persistence and evolutionary potential of forest tree species.

### 4.1 Transferability of the SSR Markers

Developing SSR markers, particularly for non‐model species, is both time‐consuming and costly (Moosavi et al. 2022). Due to the species‐specific nature of most SSR primers, their cross‐species transferability is often limited. However, EST‐SSRs, which are derived from expressed sequence tags, offer greater transferability between closely related species, making them particularly valuable for non‐model organisms (Ellis and Burke 2007; Moosavi et al. 2022).

The enhanced transferability of EST‐SSRs compared to genomic SSRs (gSSRs) is attributed to their targeting of conserved regions, which tend to be more stable across species. However, this conservation has led to lower genetic variation compared to gSSRs (Vieira et al. 2016).

Our results indicate higher transferability of EST‐SSRs (approximately 46%) compared to gSSRs (approximately 33%) in *Q. castaneifolia*, a pattern consistent with previous findings showing that EST‐derived SSRs generally transfer across related species more readily than genomic SSRs (Durand et al. 2010; Bodénès et al. 2012). Chloroplast SSR markers displayed a contrasting pattern: despite a higher amplification success rate (approximately 67%), polymorphism was limited (approximately 10%), which is consistent with the slow evolutionary rate and uniparental inheritance of chloroplast genomes (Weising and Gardner 1999; Deguilloux et al. 2003; Götz et al. 2020). These findings align with broader trends observed across the *Quercus* genus, where chloroplast markers show lower intraspecific variation but higher transferability across species, as demonstrated in large‐scale phylogeographic studies (Petit et al. 2002; Neophytou et al. 2011). The success of cross‐species amplification is influenced by evolutionary distance, with closely related taxa demonstrating higher transferability (Shekhar et al. 2021).

### 4.2 Genetic Variation Based on nSSR and cpSSR

Our results revealed a moderate level of genetic diversity in *Q. castaneifolia* populations based on nuclear SSR markers, including both EST‐derived and genomic loci (Table 1). Therefore, comparisons with other *Quercus* species were restricted to the subset of EST‐SSR loci that are shared with and were originally developed for *Q. robur* . Based on this common set of markers, the effective number of alleles (*N*_ae_ = 3.06) and standardized allelic richness (A_R_ = 5.57) observed in *Q. castaneifolia* were generally lower than the values reported for *Q. robur* , whereas expected heterozygosity was comparable (Burger et al. 2021). However, comparisons with other *Quercus* species may still be informative, and future studies including additional *Cerris* species could provide further insights. Because genomic SSRs and nonoverlapping loci were excluded from this comparison, the observed differences should be interpreted as relative patterns rather than strict quantitative contrasts. In contrast, studies using gSSRs have reported greater genetic diversity estimates, highlighting the influence of marker type on diversity assessment (Crăciunesc et al. 2016; Ellis and Burke 2007). In our study, nongenic gSSRs showed higher variation than EST‐SSRs, consistent with previous findings that noncoding intergenic regions generally harbor greater polymorphism due to reduced selective constraints (Ellis and Burke 2007).

### 4.3 Relationship Between Environmental and Genetic Variation

In the Hyrcanian forest, populations of *Q. castanifolia* are widely distributed and exhibit a broad ecological range, suggesting that natural selection may influence the genetic structure of this species. Based on our dataset, we found no evidence of isolation by distance or that site conditions impact genetic variation as a result of adaptation. None of the environmental variables (e.g., temperature, precipitation, and soil pH) and elevation showed a significant relationship with individual heterozygosity, and the Mantel test for isolation by distance was also nonsignificant. These results are consistent with the genome‐wide study of *Q. castanifolia* by Vatanparast et al. (2024), who similarly detected no significant correlation between nucleotide diversity and either longitude or elevation across the Hyrcanian range (their Figure 3). Interestingly, the same study reported significant spatial–environmental correlations with nucleotide diversity for *Acer velutinum* and *Fagus orientalis* in the same region, implying species‐specific responses to ecological gradients. Despite the differences in inferred cluster numbers (*K* = 1 for our nuclear SSR dataset and *K* = 2 for the chloroplast SSR and RADseq dataset reported by Vatanparast et al. 2024), both datasets consistently lack clear spatial or environmental patterns. This concordant absence reinforces the interpretation that, in *Q. castanifolia*, extensive pollen‐mediated gene flow has homogenized nuclear genetic variation across environmental gradients, masking potential signatures of local adaptation. Comparative studies in European oaks suggest similar patterns of gene flow and limited environmental structuring. Homolka et al. (2013) observed low population differentiation in drought‐adapted *Q. petraea* and *Q. robur* , despite some allele‐environment correlations. Neophytou et al. (2015) reported high within‐population diversity and weak spatial structuring across multiple European oak species. Derory et al. (2010) highlighted locus‐specific adaptive variation rather than genome‐wide differentiation. These studies support the interpretation that extensive pollen‐mediated gene flow can homogenize nuclear genetic variation, consistent with our findings in *Q. castanifolia*.

### 4.4 Genetic Structure Based on nSSR and cpSSR

PCoA and STRUCTURE analyses of nSSR markers revealed no significant genetic or geographic population structure, irrespective of elevation or longitudinal gradients (Figures S2a and S3). The low genetic differentiation (*F*_ST_ = 0.019) indicates efficient gene flow, which aligns with patterns observed in many temperate tree species (Porth and El‐Kassaby 2014). The observed lack of genetic structure can be attributed to the high dispersal capacity of *Quercus* species, which are primarily wind‐pollinated and capable of long‐distance gene flow (Schueler & Schlünzen 2006). Furthermore, acorn dispersal by animals, particularly jays and rodents, is known to facilitate seed movement across extensive geographic ranges, contributing to genetic homogenization among populations (González‐Rodríguez and Oyama 2005; Coelho et al. 2006). Nonsignificant inbreeding coefficients (*F*_IS_) further suggest a near‐panmictic reproduction system, where mating occurs randomly among individuals, thereby reducing localized genetic differentiation. However, genome‐wide SNP data by Vatanparast et al. (2024) revealed two weakly differentiated genetic clusters across the Hyrcanian range, although the overall *F*_ST_ remained low (0.029). This apparent discrepancy likely reflects differences in marker resolution: their dataset included thousands of loci, providing higher power to detect subtle structure that might remain undetected in SSR datasets with fewer loci. Together, both studies point to predominantly high connectivity, but also suggest that minor east–west sub‐structuring may exist at finer genomic scales.

In contrast, cpSSR data indicated a more pronounced genetic differentiation, with distinct eastern and western clusters (Figure S3). Among the four hydro‐regions analyzed, western populations (especially Talesh–Mardab) exhibited the highest haplotype richness, including several private haplotypes (H1, H4, and H6_H7) confined to the western Hyrcanian region. Eastern populations shared common haplotypes (H2–H3) with much higher frequencies (> 70%). The Lahijan–Nur populations in central Mazandaran showed a mixture of western and eastern haplotypes, consistent with their intermediate geographic position. Overall, this west–east pattern supports restricted seed‐mediated dispersal and historical differentiation across the Hyrcanian range.

The occurrence of haplotype H5 exclusively in Haraz‐Neka, and its absence in adjacent populations may suggest a recent introduction, possibly facilitated by human‐mediated seed or seedling movement (e.g., through plantation or transportation activities). However, additional sampling and genomic data would be required to confirm this hypothesis. Unlike nuclear markers, which show biparental inheritance, chloroplast markers are typically maternally inherited (via seeds) in angiosperms, making them more sensitive to historical and geographical barriers to gene flow. This differentiation at maternally inherited cpSSRs highlights the contrasting patterns of gene flow via pollen and seeds, emphasizing the importance of seed dispersal constraints in shaping the observed genetic structure. The clear structure observed in cpSSRs suggests that past climatic events or geographic barriers may have influenced seed‐mediated gene flow. The Caspian Sea has experienced substantial sea‐level fluctuations throughout the Quaternary due to regional climatic and hydrological changes (Rychagov 1997; Leroy and Arpe 2007). One plausible explanation for this differentiation is the impact of historical Caspian Sea transgressions during the Late Pleistocene, which likely acted as a barrier to gene flow, limiting seed dispersal between eastern and western populations (Kazancı et al. 2004; Gelfan et al. 2024). These fluctuations in sea level, along with changes in forest cover, may have contributed to the formation of isolated refugia, leading to the observed differentiation in chloroplast haplotypes.

Similar patterns of east–west genetic structure have been reported in other species of the Hyrcanian forest, including both plants and animals, underscoring the potential influence of shared historical biogeographic processes (Salehi Shanjani et al. 2010; Erichsen et al. 2018; Maharramova et al. 2018; Alipour et al. 2021; Najibzadeh et al. 2018; Ahmadi et al. 2018). The presence of multiple private haplotypes in the Talesh–Mardab population further indicates that this region may have functioned as a refugium or a center of historical genetic diversity for *Q. castaneifolia*. Although cpSSR analyses reveal clear east–west structuring, the small number of polymorphic chloroplast loci (*n* = 4) limits the resolution of seed‐mediated genetic patterns; therefore, targeted chloroplast sequencing or SNP‐based chloroplast analyses in Talesh–Mardab are recommended. In contrast, the genetic uniformity observed in the Qarasu–Gorgan hydro‐region, characterized by the detection of only a single haplotype, suggests a potential genetic bottleneck or founder effect, possibly resulting from limited seed dispersal or historically influenced genetic diversity contractions. The occurrence of both western and eastern haplotypes in Lahijan–Nur indicates mixed seed‐mediated genetic influences from both eastern and western populations.

The contrasting patterns observed between nSSR and cpSSR markers highlight the importance of considering both pollen‐ and seed‐mediated gene flow in understanding the evolutionary history of *Q. castaneifolia*. While pollen dispersal appears to maintain genetic connectivity among populations, seed‐mediated gene flow has been more limited, resulting in the observed chloroplast genetic structure. These findings carry important conservation implications, particularly for maintaining genetic diversity in regions that exhibit unique haplotypes. Conservation strategies should prioritize genetically distinct populations such as Talesh–Mardab, while also explicitly incorporating the two genome‐wide clusters reported by Vatanparast et al. (2024). Crucially, our UNICOR connectivity modeling identified two spatially explicit core areas—one in the western Hyrcanian (Talesh–Mardab) and one in the eastern Hyrcanian (Qarasu–Gorgan) that broadly align with the east–west genomic differentiation. Prioritizing protection of representative populations from both genomic clusters and from both connectivity cores, and ensuring these areas are incorporated into protected‐area networks or ecological corridors, will help conserve both historic (seed‐mediated) and contemporary (pollen‐mediated, genome‐wide) genetic variation.

### 4.5 Core Habitats and Connectivity Corridors as Key Areas of Chloroplast Diversity Outside Protected Zones

The connectivity analysis in UNICOR revealed two main core habitats—Talesh–Mardab in the west and Qarasu–Gorgan in the east—linked by a narrow corridor extending through Lahijan–Nur and Haraz–Naka. Notably, the spatial distribution of chloroplast haplotypes closely overlapped with these connectivity cores, particularly in the western region, where multiple private haplotypes were concentrated. This pattern indicates that areas identified as highly connected by UNICOR correspond to zones of elevated chloroplast haplotype diversity, likely reflecting long‐term habitat stability. In contrast, eastern populations exhibited low haplotype diversity and no private haplotypes, consistent with the absence of long distance seed dispersal across regions. Importantly, none of the unique or regionally restricted haplotypes occurred within currently protected areas (Figure 2), implying that the existing conservation network fails to encompass key reservoirs of chloroplast diversity. This mismatch highlights a critical gap between genetic and administrative conservation priorities. Integrating these genetically important sites—especially the western core around Talesh–Mardab—into future protection schemes or ecological corridors would therefore be essential for maintaining seed‐mediated diversity.

### 4.6 Comparison With Genomic Data

Our nuclear SSR results reveal substantial nuclear genetic diversity and weak large‐scale population structure in *Q. castaneifolia*. These patterns are consistent with recent genome‐wide SNP data reported by Vatanparast et al. (2024). Together, these independent datasets support the interpretation that pollen‐mediated gene flow has been and continues to be an important homogenizing force in *Q. castaneifolia* across the Hyrcanian forests.

Importantly, our cpSSR results (seven chloroplast haplotypes with several private haplotypes concentrated in Talesh–Mardab) add a complementary perspective: while genome‐wide SNPs and nSSRs indicate contemporary connectivity by pollen‐mediated gene dispersal, the localized cpDNA structure points to historical seed‐mediated isolation and potential refugial persistence in the western Hyrcanian (Talesh–Mardab). Taken together, the concordance of nuclear markers across marker types and the contrasting signal from maternally inherited markers support a scenario of long‐term refugial retention of cp haplotypes combined with ongoing pollen‐mediated homogenization.

**FIGURE 5:**
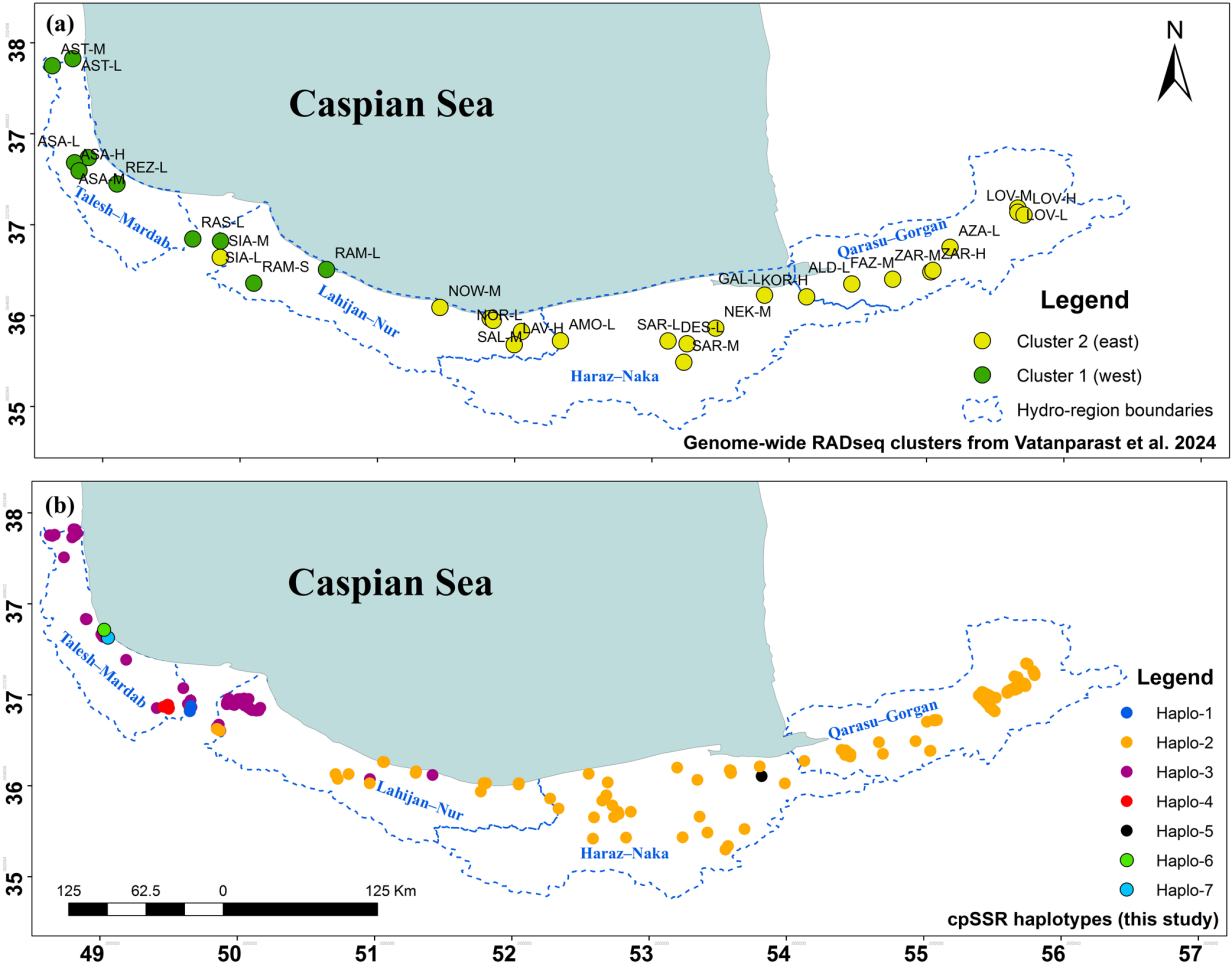
Spatial distribution of genome‐wide SNP clusters (*K* = 2) identified by Vatanparast et al. (2024) across the Hyrcanian forests (a), shown alongside cpSSR haplotype distributions from this study (b).

To directly compare our cpSSR patterns with genome‐wide structure, we overlaid the predominant RADseq clusters (*K* = 2) from Vatanparast et al. (2024) (Figure 5a) and cpSSR haplotype (Figure 5b). The SNP‐based clusters displayed a clear west–east split, with western sites largely assigned to cluster 1 and eastern sites to cluster 2 (Figure 5a). This pattern corresponds strongly with the cpSSR haplotype distribution observed in our study (Figure 5b). Rare mismatches between SNP clusters and local cpSSR haplotypes—particularly in the western region—suggest isolated cases of long‐distance seed dispersal (Figure 5a,b). While in Lahijan–Nur the SNP analysis detected individuals belonging to both genomic clusters (Figure 5a), our chloroplast data similarly showed the co‐occurrence of Haplo‐2 and Haplo‐3 in this region (Figure 5b). This concordance suggests that the Lahijan–Nur hydro‐region represents a genetic transition zone where eastern and western lineages meet and occasionally intermix (Figure 5a,b).

### 4.7 Implications for Conservation

The findings of this study have important conservation implications for *Q. castaneifolia* and the Hyrcanian forest ecosystem. The Talesh–Mardab population, which harbors several unique chloroplast haplotypes and the highest haplotypic richness, should be prioritized for conservation. These private haplotypes likely reflect local persistence under stable environmental conditions rather than historical evolutionary divergence, highlighting the need to preserve these areas as reservoirs of genetic diversity.

Crucially, none of the unique haplotypes were found within existing protected areas, revealing a gap between genetic and administrative conservation coverage. Integrating these genetically valuable regions—particularly the western core around Talesh–Mardab and the connecting corridor through Lahijan–Nur and Haraz–Naka into future conservation and management plans would strengthen the long‐term protection of the species' genetic resources.

Moderate nuclear genetic diversity across populations suggests that *Q. castaneifolia* still maintains sufficient variation to cope with short‐term environmental pressures, although ongoing habitat loss could threaten this potential. Maintaining both habitat connectivity and effective population sizes will be critical for sustaining gene flow and mitigating genetic erosion.

Overall, our results provide a spatially explicit framework for integrating genetic and landscape connectivity data into conservation planning, enabling more effective protection of both maternal and nuclear genetic diversity in *Q. castaneifolia* throughout the Hyrcanian forests. Building on these findings, future studies could implement provenance trials and genotype–phenotype assessments across the Hyrcanian range to evaluate phenotypic diversity and the adaptive potential of populations. Such research would provide practical guidance for selecting resilient populations and refining conservation strategies in response to changing climatic conditions.

## 5 Conclusion

While the previous SNP‐based study by Vatanparast et al. (2024) consistently reported high nuclear genetic diversity and very low genetic differentiation across Hyrcanian populations, our results using nSSR markers confirm this pattern, showing similarly high diversity and weak population structure. In addition, cpSSR markers reveal localized cpDNA haplotype variation in the western Hyrcanian range, which shows a general correspondence with the SNP‐based clusters, suggesting regions where maternal lineages of different origins may co‐occur.

Overall, integrating cost‐effective microsatellite markers with landscape connectivity modeling can provide preliminary, spatially explicit guidance for conservation planning. Our results suggest that even with a limited number of markers, it may be possible to identify potential corridors for gene flow, which could help prioritize conservation actions. However, these suggestions should be considered provisional and need to be validated by higher‐resolution genomic studies to determine whether the localized cpDNA patterns are reflected across the genome.

## Author Contributions

**Gilda Shahnaseri:** data curation (equal), formal analysis (equal), software (equal), validation (equal), visualization (equal), writing – original draft (equal). **Mansoureh Malekian:** conceptualization (equal), supervision (equal), writing – review and editing (equal). **Markus Müller:** formal analysis (equal), resources (equal), software (equal). **Oliver Gailing:** funding acquisition (equal), methodology (equal), project administration (equal), writing – review and editing (equal).

## Funding

The marker analyses were funded through start‐up funds of the University of Göttingen to OG. GSH was awarded a Max G. Huber Scholarship by the DAAD‐Stiftung.

## Conflicts of Interest

The authors declare no conflicts of interest.

## Supporting information


**Figure S1:** Locations of the sampling sites along the (a), annual precipitation gradient (b), and soil‐pH gradient (c) for the Hyrcanian Forest area.
**Figure S2:** The PCoA of nSSR (a) and cpSSR (b) markers for the *Q. Castaneifolia* samples.
**Figure S3:** Bar plot of admixture coefficients at different *K* values for each individual based on nSSRs (a) and cpSSRs (b).
**Figure S4:** Plot of the K and Mean LnP(K) values obtained from the SRUCTURE SELECTOR for nSSR (a) and cpSSR (b) markers.

**Table S1:** List of nuclear SSRs (nSSRs), including EST‐SSRs and genomic SSRs, tested for amplification and polymorphism. MM, Monomorphic; NA, No amplification; PA, Poor amplification; PM, Polymorphic.
**Table S2:** List of chloroplast SSRs (cpSSRs) tested for amplification and polymorphism. MM, Monomorphic; NA, No amplification; PA, Poor amplification; PM, Polymorphic.
**Table S3:** Based on MICRO‐CHECKER analysis, null allele frequency was estimated for 11 microsatellite loci.
**Table S4:** Genetic variation values across all samples for each nSSR marker.
**Table S5:** Genetic variation at four cpDNA loci for each marker in the four subpopulations.

**Data S1:** Supporting information.

## Data Availability

All the required data are uploaded as Supporting Information.
