# Supplementary material for: Conservation Hotspots of Quercus castaneifolia Revealed Through the Integration of Genetic Diversity and Landscape Connectivity
Source: Ecol Evol. 2026 Apr 17;16(4):e73393. doi: 10.1002/ece3.73393 (PMC13090102; doi:10.1002/ece3.73393)
Supplement: Supplementary file 1 — Figure S1: Locations of the sampling sites along the (a), annual precipitation gradient (b), and soil‐pH gradient (c) for the Hyrcanian Forest area. Figure S2: The PCoA of nSSR (a) and cpSSR (b) markers for the Q. Castaneifolia samples. Figure S3: Bar plot of admixture coefficients at different K values for each individual based on nSSRs (a) and cpSSRs (b). Figure S4: Plot of the K and Mean LnP(K) values obtained from the SRUCTURE SELECTOR for nSSR (a) and cpSSR (b) markers. [file ECE3-16-e73393-s001.docx]

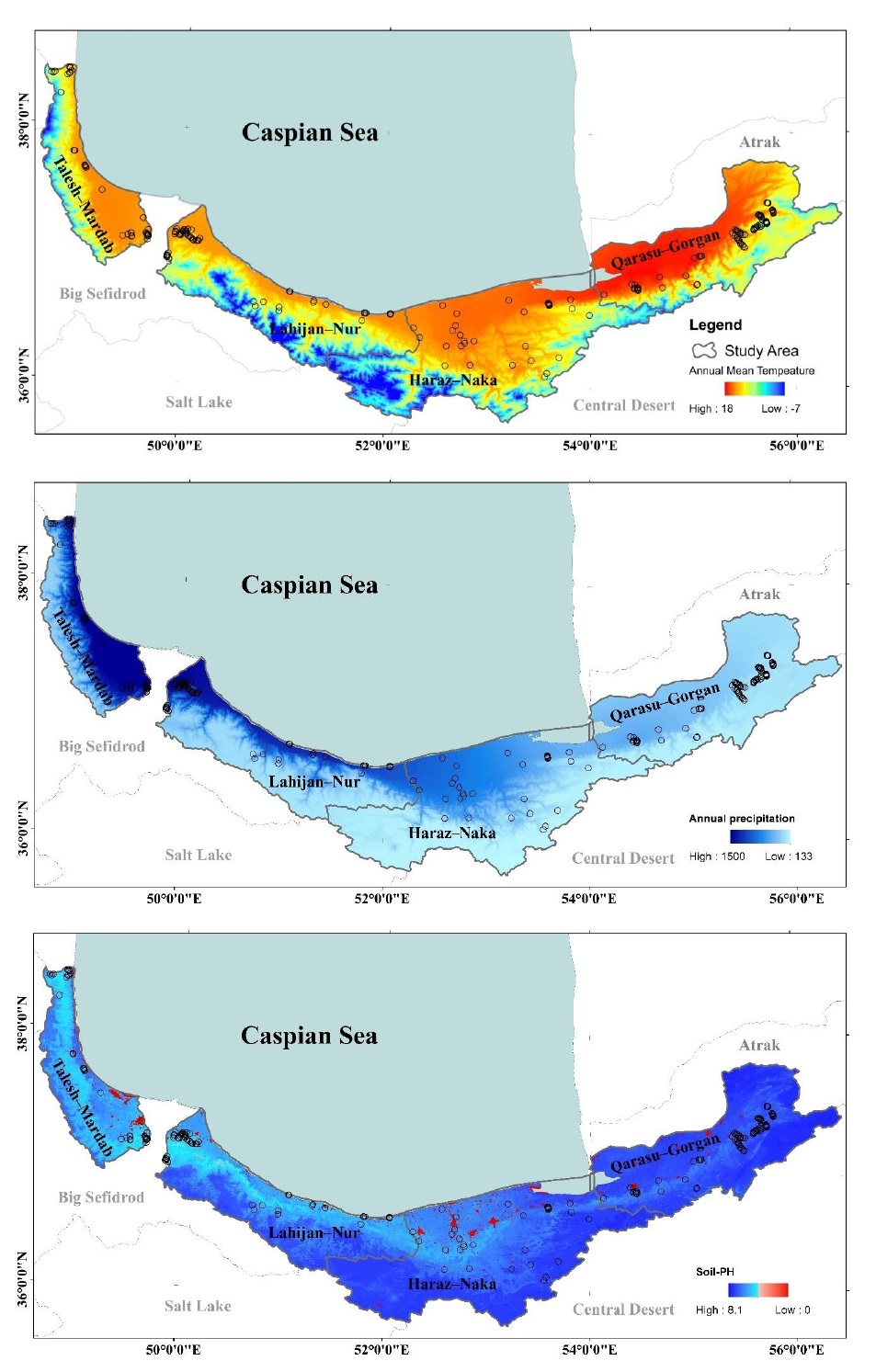


(c)

(b)

(a)

**Figure S1.** Locations of the sampling sites along the (a), annual precipitation gradient (b), and soil-pH gradient (c) for the Hyrcanian Forest area.

(a)

(b)

**Figure S2.** The PCoA of nSSR (a) and cpSSR (b) markers for the *Q. Castaneifolia* samples.

| 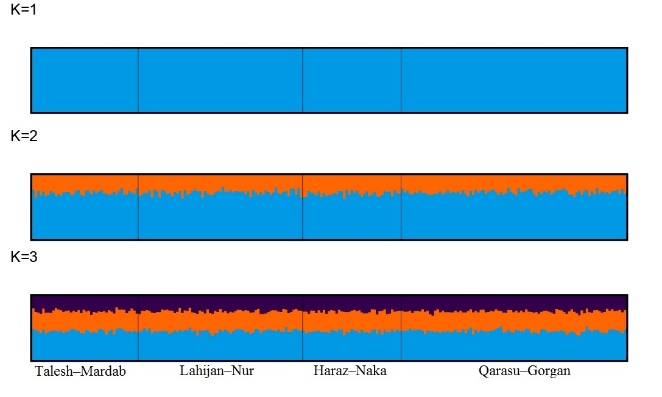(a) | 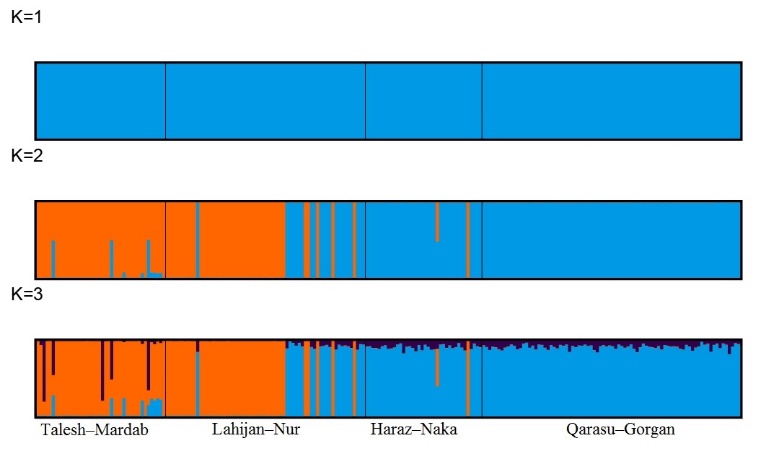 (b) | |
| --- | --- | --- |
| **Figure S3.** Bar plot of admixture coefficients at different K values for each individual based on nSSRs (a) and cpSSRs (b). | |  |

**Genetic variation across populations using cpSSRs**

| 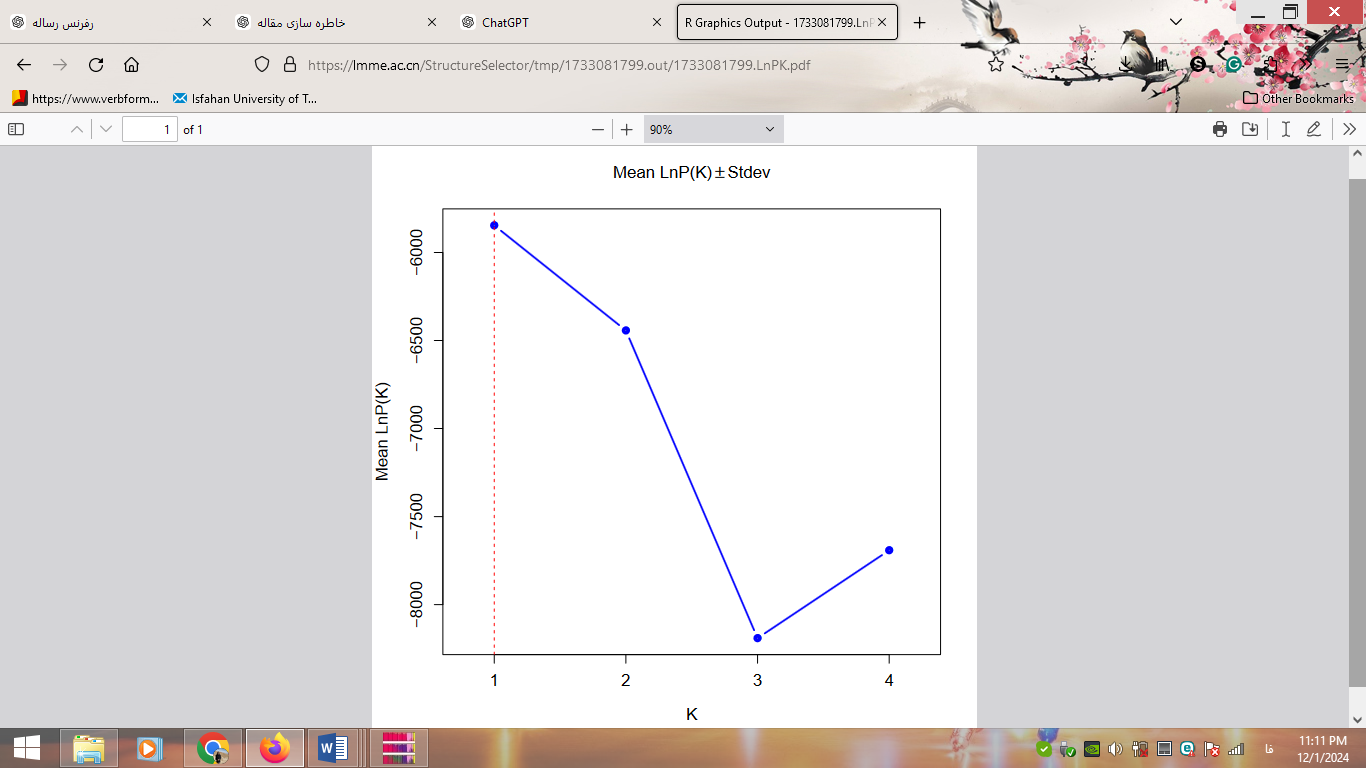  (a)**a** | 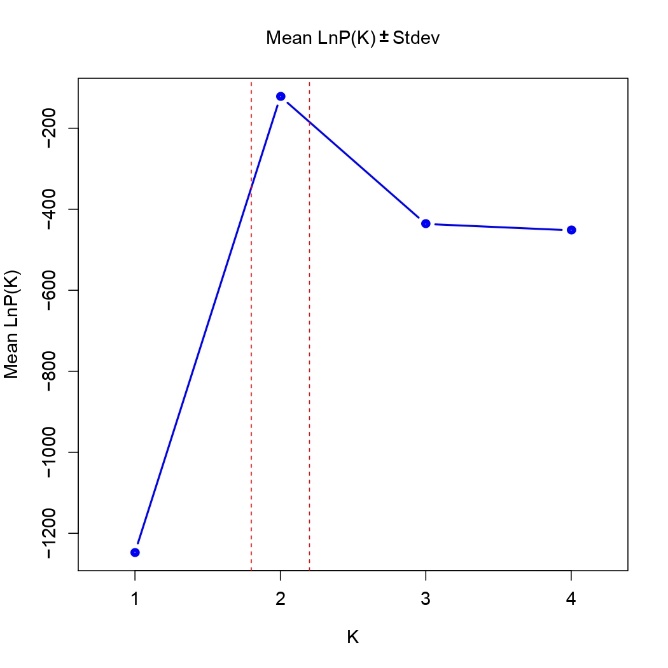  (b) |
| --- | --- |
| **Figure S4.** Plot of the K and Mean LnP(K) values obtained from the SRUCTURE SELECTOR for nSSR (a) and cpSSR (b) markers. | |
