# Supplementary material for: Conservation Hotspots of Quercus castaneifolia Revealed Through the Integration of Genetic Diversity and Landscape Connectivity
Source: Ecol Evol. 2026 Apr 17;16(4):e73393. doi: 10.1002/ece3.73393 (PMC13090102; doi:10.1002/ece3.73393)
Supplement: Supplementary file 2 — Table S1: List of nuclear SSRs (nSSRs), including EST‐SSRs and genomic SSRs, tested for amplification and polymorphism. MM, Monomorphic; NA, No amplification; PA, Poor amplification; PM, Polymorphic. Table S2: List of chloroplast SSRs (cpSSRs) tested for amplification and polymorphism. MM, Monomorphic; NA, No amplification; PA, Poor amplification; PM, Polymorphic. Table S3: Based on MICRO‐CHECKER analysis, null allele frequency was estimated for 11 microsatellite loci. Table S4: Genetic variation values across all samples for each nSSR marker. Table S5: Genetic variation at four cpDNA loci for each marker in the four subpopulations. [file ECE3-16-e73393-s002.docx]

|  | |  | | **Table S1.** List of nuclear SSRs (nSSRs), including EST-SSRs and genomic SSRs, tested for amplification and polymorphism. Abbreviations: PM = Polymorphic; MM = Monomorphic; PA = Poor Amplification; NA = No Amplification. | | | | | | | |
| --- | --- | --- | --- | --- | --- | --- | --- | --- | --- | --- | --- |
| No. | Primer | | Location | | Repeat motif | Multiplex set | Product size | Sequence (5’–3’) | Functional annotation | Product quality^a^ | Reference |
| 1 | FIR013 | | EST-SSR | | (CAG)_5_ |  | 129-150 | F:CGGGGAGGTTGATGAGTATT  R:AACACTGTCACCCCCATAGC | constans-like protein COL  (flowering time) | MM | Durand et al. (2010) |
| 2 | FIR028 | | EST-SSR | | (TC)_8_ | 2 | 200-250 | F:GGAAGAGTGTTCGGAAAGCA  R:CCAGCTCCTCCACAATAGCA | tropinone reductase, putative (drought stress) | PM |  |
| 3 | FIR035 | | EST-SSR | | (AT)_6_ | 2 | 160-190 | F:GCTAAGGTTCCGTGTTCCAA  R:GGCCAGCAACTAAACCAAGA | chaperone protein dna J,  chloroplast, putative (heat  shock protein, stress) | PM |  |
| 4 | FIR043 | | EST-SSR | | (TC)_9_ |  | 114-146 | F:PETTTCTCCATTTCACACGCTTC  R:ACGACATCGTTTTGGAGCTT | EBP1 (regulates cell growth) | PA |  |
| 5 | FIR104 | | EST-SSR | | (GGT)_7_ |  | 207-225 | F:TTAACTCGGTTTGCGACTCA  R:AGCACGTGACTCGACCTGTA | r^2^r^3^-myb transcription factor | PA |  |
| 6 | FS_C2361 | | EST-SSR | | (GAA)_8_ |  | 193-196 | F:AGGTCCTTCAGTTTGGGAGC  R:ATTCCCATGCATCAAAATCC | Light-harvesting complex-like  protein OHP2 | MM | Burger et al. (2018) |
| 7 | FS_C2660 | | EST-SSR | | (GAG)_8_ | 3 | 215-240 | F:AGCAGAATTCGCCAAGTGAT  R:TGCCTTTGCATTCTCCTCTT | Eukaryotic translation initiation factor 5B-like | PM |  |
| 8 | FS_C2791 | | EST-SSR | | (GA)_5_ |  | 287-302 | F:CGAAACAGAGAGAACCCAAGA  R:CTTCAAACATCCAGCGTTGA | 50S ribosomal protein L13,  chloroplastic | MM |  |
| 9 | FS_C8183 | | EST-SSR | | (AGC)_6_ | 3 | 190-213 | F:TATTCAACCACAGCTGCCTG  R:ACAGCTGCCTCTGTGGATCT | auxin response factor | PM |  |
| 10 | GOT040 | | EST-SSR | | (GA)_11_ | 4 | 220-240 | F:AAGGCACTCGTCGCTTTCTA  R:ACCGATTTGAAGCTCGAGAA | 40s ribosomal protein s16 | PM | Durand et al. (2010) |
| 11 | PIE040 | | EST-SSR | | (TTC)_8_ | 4 | 160-220 | F:GTGAGAGAGAGAGAGACAAAGAAGAAAAA  R:AAATTCTCCGCCACATTGAG | Retrotransposon-related protein | PM |  |
| 12 | PIE102 | | EST-SSR | | (AG)_12_ |  | 130-150 | F:ACCTTCCATGCTCAAAGATG  R:GCTGGTGATACAAGTGTTTGG |  | MM |  |
| 13 | PIE125 | | EST-SSR | | (GGAAGC)_3_ | 4 | 140-180 | F:AATACAAATCGCAGGAGGTG  R:CTAACCCATCGTTCATGGAG | chaperone protein dna J, chloroplast, putative (heat shock protein, stress) | PM |  |
| 14 | PIE215 | | EST-SSR | | (GAG)_6_ |  | 215-227 | F:ACGAAATGGAGCTGTTGACC  R:TCTCCTTCTCTTCTGCCATGA |  | MM |  |
| 15 | PIE223 | | EST-SSR | | (GGT)_8_ | 3 | 88-108 | F:AGAAGCCCAACACGGCTAC  R:AGCAAAACACAAACGCACAA |  | PM |  |
| 16 | PIE227 | | EST-SSR | | (TGG)_8_ |  | 140-175 | F:ACCATGATCTGGGAAGCAAC  R:AAGGGCTTGGTTGGGTTAGT |  | MM |  |
| 17 | PIE239 | | EST-SSR | | (AT)_12_ |  | 80-115 | F:CAACAAATGGCTCAACAGTG  R:CCCATTTGGTAGCAAAGAGTC |  | MM |  |
| 18 | PIE267 | | EST-SSR | | (AG)_11_ |  | 85-105 | F:CCAACCATCAAGGCCATT  R:GTGCGAACAGATCCCTTGTC |  | NA |  |
| 19 | PIE271 | | EST-SSR | | (TC)_11_ |  | 181-230 | F:CACACTCACCAACCCTACCC  R:CACACTCACCAACCCTACCC |  | PA |  |
| 23 | VIT023 | | EST-SSR | | (ATA)_6_ |  | 117-120 | F:AATGCGAACGACATGAACAA  R:CTCTCGTCGGAGACTCAACC | ap2 erf domain-containing transcription factor | MM |  |
| 24 | VIT107 | | EST-SSR | | (TA)_13_ | 2 | 126-138 | F:TGATCACAGATTGGAGCTTAACA  R:CCCCCACTTAGGAAAGAAGC | light-harvesting complex i protein lhca2 | PM |  |
| 20 | QrC0057 | | EST-SSR | | (AAG)_7_ | 3 | 126-146 | F:CCGACCTTGTTGATTGTTCC  R:TATTGATCCTATCGGAGGCG | Vacuolar protein sorting associated  protein 60.1 | PM | Müller & Gailing (2018) |
| 21 | QrC0332 | | EST-SSR | | (CCT)_5_ | 3 | 150-180 | F:AATATCAAATCGGCCAGCAG  R:GTGGTGGACCTGTGCCATAC | DEAD-box ATP-dependent  RNA helicase 20 | PM |  |
| 22 | Qr1423 | | EST-SSR | | (CAC)_6_ |  | 276-282 | F:TCCCTTCTCGTTTCACCATC  R:TGCACCATACGGATTGAAAG | Galactan beta-1,4-  galactosyltransferase GALS3-  like | MM |  |
| 25 | quru-CA-2P24 | | gSSR | | (CA)_14_ | 1 | 117-175 | F:GCAAGAGATCACACACAAACTAGC  R:CTTTGGGTTCACCAAACAGC |  | PM | Sullivan et al. 2013 |
| 26 | quru-CA-3A05 | | gSSR | | (CA)_12_(CT)_2_ | 1 | 130-180 | F:AACGTGACCTCTCTCACAGC  R:AGTGCTGGAGTGCTCATGG |  | PM |  |
| 27 | quru-GA-3D15 | | gSSR | | (CA)_15_ |  | 210-217 | F:GGTGGTGGCAGATACACTGG  R:GACTCAGACAACCAACTTCAGG |  | MM |  |
| 28 | quru-GA-1P10 | | gSSR | | (TG)_12_GCC(TG)_3_ |  | 234-270 | F:ATTTCTGATGCAGGGTGTCG  R:TAGGCCAAGGACCAGAGACC |  | MM | Aldrich et al. (2002) |
| 29 | quru-GA-0C11 | | gSSR | | (GA)_15_ |  | 201-233 | F:TCCCCAAATTCAGGTAGTGT  R:ICATACCCAGCTCCCATGACCA |  | MM |  |
| 30 | quru-GA-0E09 | | gSSR | | (GA)_16_ |  | 174-250 | F:TGCCATCCCTATACACAACCA  R:CCTCCATCACAAAGTTGCC |  | NA |  |
| 31 | quru-GA-1C06 | | gSSR | | (GA)_29_ | 2 | 240-270 | F:CAAATAAATATTGTGGGGTTCA  R:GGAGGGGATCCGGAAAA |  | PM |  |
| 32 | quru-GA-1F07 | | gSSR | | (GA)_22_ |  | 294-352 | F: CAAATAAATATTGTGGGGTTCA R:GGGTGGATTGGGTTTCTACCTA |  | PA |  |
| 33 | quru-GA-2F05 | | gSSR | | (GA)_21_ |  | 317-281 | F:CCGCTTCGTGACGATTATTC  R:GAGGTTTGGAGGAGAGATCATTCT |  | NA |  |

|  | | **Table S2.** List of chloroplast SSRs (cpSSRs) tested for amplification and polymorphism. Abbreviations: PM = Polymorphic; MM = Monomorphic; PA = Poor Amplification; NA = No Amplification. | | | | | | | |
| --- | --- | --- | --- | --- | --- | --- | --- | --- | --- |
| No. | Primer | | Location | Repeat motif | Product size | Sequence (5’–3’) | Functional annotation | Product quality^a^ | Reference |
| 1 | QRcp01 | | cpSSR | (T)_10_ | 235-245 | F:CGTTTAATTAGATCGGGTAATCGT  R:GCAGCATGTCGTATCAATGTGG |  | MM | Götz & Gailing 2022 |
| 2 | QRcp02 | | cpSSR | (A)_11_ | 185-195 | F:AGTTTCTGTTTTCCTTGCTTGATTT  R:CGAATCCCTCTCTTTCCGCT |  | NA |  |
| 3 | QRcp07 | | cpSSR | (A)_16_ | 200-210 | F:ACCTTTCGGGGAGTGATTGG  R:TCCCTCTTCCCAGATTTCCA |  | NA |  |
| 4 | QRcp011 | | cpSSR | (A)_11_ | 355-365 | F:CGAATGGAGGCCCTTATTTTCA  R:CCACTTCCGAATGGTATGCT | Chloroplast ribosomal protein | NA |  |
| 5 | QRcp013 | | cpSSR | (T)_14_ | 245-255 | F:TACCAAACGATTGGGATGCT  R:AGAAATGCATGAAAGAGCCCC |  | MM |  |
| 6 | QRcp014 | | cpSSR | (A)_9_ | 162-172 | F:GGGCCCTCTCTTTATACCTCT  R:TCGCTTTCGAGCCCTTACTT |  | MM |  |
| 7 | QRcp017 | | cpSSR | (A)_16_ | 175-190 | F:CCGCGAGACCAGAAAGGG  R:TCCCTCTTCCCAGATTTCCA |  | NA |  |
| 8 | QRcp024 | | cpSSR | (T)_9_ | 196-210 | F:AACGAGTCACACACTAAGCA  R:AGCCTAAGCCTTTATTTGACTTG |  | MM |  |
| 9 | QRcp028 | | cpSSR | (T)_14_ | 188-196 | F:TAGATCGTTCTGCAAAGCCC  R:TCGTTGGACAACCACTATGTCT |  | PM |  |
| 10 | QRcp029 | | cpSSR | (T)_12_ | 196-205 | F:GGCAATTGCGATGGCTTCTT  R:TTCGGGGGAAACCACATCAC |  | PM |  |
| 11 | QRcp030 | | cpSSR | (T)_14_ | 245-255 | F:TACCAAACGATTGGGATGCT  R:AGAAATGCATGAAAGAGCCCC |  | NA |  |
| 12 | QRcp031 | | cpSSR | (T)_12_ | 130-140 | F:CGGAGGACTCTTCTGAACAAACA  R:ATCCAATGCTGAATCGATGACCT |  | MM |  |
| 13 | QRcp033 | | cpSSR | (T)_11_ | 180-195 | F:CCAAGGAGAAGATGCGGGTT  R:GGTGGGCAGGAGGAAAAGAA |  | NA |  |
| 14 | QRcp034 | | cpSSR | (A)_16_ | 95-110 | F:CTTCTTTTCCTCCTGCCCACC  R:ATGGAGGTTTGACTCCGGTAA |  | NA |  |
| 15 | QRcp041 | | cpSSR | (T)_9_ | 100-110 | F:GAAGCACGAACCAACCCCT  R:TTGCTCGAAAGGAATGCGGT |  | MM |  |
| 16 | μdt1 | | cpSSR | (A)_11_ | 86 | F:ATCTTACACTAAGCTCGGAA  R:TTCAATAACTTGTTGATCCC |  | MM | Deguilloux et al. 2003 |
| 17 | μdt3 | | cpSSR | (A)_11_ | 126 | F:TGTTAGTAATCCTTTCGTTT  R:AGGTATAAAGTCTAAGGTAA |  | NA |  |
| 18 | μdt4 | | cpSSR | (A)_9_ | 147 | F:GATAATATAAAGAGTCAAAT  R:CCGAAAGGTCCTATACCTCG |  | MM |  |
| 19 | μdt5 | | cpSSR | (A)_8_ | 159 | F:TAAATCTGGAAATCTGGGAA  R:TTGATACATAGACTTGCCAA |  | NA |  |
| 20 | μdt6 | | cpSSR | (A)_7_ | 83 | F:CTAGATGGATCATTAGCAAA  R:TCTGATATATTTTTACCGCT |  | NA |  |
| 21 | μcd1 | | cpSSR | (G)_7_ | 110 | F:ATAACTTAACAAACTTTTCCA  R:GGATTTACATCATTTTAGAGA |  | NA |  |
| 22 | μcd2 | | cpSSR | (T)_7_ | 152 | F:AATAGACTATCGTCCCATTAT  R:TGACCTTCATTTCATTATCAT |  | NA |  |
| 23 | μcd3 | | cpSSR | (T)_7_ | 165 | F:GTCCTTACATTTTCCCCTTCA  R:AGAGGTGAAACCAAAAACAAA |  | NA |  |
| 24 | μcd4 | | cpSSR | (T)_12_ | 97 | F:TTATTTGTTTTTGGTTTCACC  R:TTTCCCATAGAGAGTCTGTAT |  | PM |  |
| 25 | μcd5 | | cpSSR | (A)_8_ | 77 | F:CCCCCGGATCTCTGTCAACTG  R: TAATAAACGAGAATCACATAA |  | NA |  |
| 26 | μcd6 | | cpSSR | (T)_7_ | 91 | F:AAAAATTATGTGATTCTCGTT  R:TATGAGAATCCTGGTATCCAC |  | NA |  |
| 27 | μcd7 | | cpSSR | (T)_9_ | 86 | F:AATTCTGCTATTTGTTCTCCC  R:CAGTCCGACGGATCCAAATAC |  | NA |  |
| 28 | μcd8 | | cpSSR | (T)_7_ | 81 | F:TCTAGGAATTAGAACCGTAAG  R:CTATTTAATTTGTGTTGATTG |  | NA |  |
| 29 | μkk1 | | cpSSR | (T)_7_ | 110 | F:CGATCAACCTCTACTCTTACT  R:GTTATGAGACCTTGGAAATGG |  | NA |  |
| 30 | μkk2 | | cpSSR | (T)_9_ | 129 | F: TTAACACAAGAAAGCCGAAGT  R:TTTATCGGATTTTGAGATTAT |  | NA |  |
| 31 | μkk3 | | cpSSR | (T)_10_ | 99 | F:TTAGATCGGGTAATCGTTCAA  R:AAGTGAATAAATGGATAGAGC |  | NA |  |
| 32 | μkk4 | | cpSSR | (T)_9_ | 114 | F:TTGTTTACCTATAATTGGAGC  R:TAGCGGATCGGTTCAAAACTT |  | NA |  |
| 33 | ccmp1 | | cpSSR | (T)_10_ | 139 | F:CAGGTAAACTTCTCAACGGA  R:CCGAAGTCAAAAGAGCGATT |  | MM | Weising & Richard 1999 |
| 34 | ccmp2 | | cpSSR | (A)_11_ | 227-228 | F:GATCCCGGACGTAATCCTG  R:ATCGTACCGAGGGTTCGAAT |  | MM |  |
| 35 | ccmp3 | | cpSSR | (T)_11_ | 112 | F:CAGACCAAAAGCTGACATAG  R:GTTTCATTCGGCTCCTTTAT |  | MM |  |
| 36 | ccmp4 | | cpSSR | (T)_13_ | 115-116 | F:AATGCTGAATCGAYGACCTA  R:CCAAAATATTBGGAGGACTCT |  | MM |  |
| 37 | ccmp5 | | cpSSR | (C)_7_(T)_10_  (T)_5_C(A)_11_ | 121 | F:TGTTCCAATATOTCTTGTCATTT  R:AGGTTCCATCGGAACAATTAT |  | MM |  |
| 38 | ccmp6 | | cpSSR | (T)_5_C(T)_17_ | 103 | F:CGATGCATATGTAGAAAGCC  R:CATTACGTGCGACTATCTCC |  | PM |  |
| 39 | ccmp7 | | cpSSR | (A)_13_ | 133 | F:CAACATATACCACTGTCAAG  R:ACATCATTATTGTATACTCTTTC |  | MM |  |
| 40 | ccmp8 | | cpSSR | (T)_6_C(T)_14_ | 77 | F:TTGGCTAСТСТААССTTCCC  R:TTCTTTCTTATTTCGCAGDGAA |  | NA |  |
| 41 | ccmp9 | | cpSSR | (T)_10_ | 98 | F:GGATTTGTACATATAGGACA  R:CTCAACTCTAAGAAATACTTG |  | NA |  |
| 42 | ccmp10 | | cpSSR | (T)_14_ | 103 | F:TTTTTTTTTAGTGAACGTGTCA  R:TTCGTCGDCGTAGTAAATAG |  | MM |  |

| **Table S3.** Based on MICRO-CHECKER analysis, null allele frequency was estimated for 11 microsatellite loci. | | | | | | |
| --- | --- | --- | --- | --- | --- | --- |
| Locus name | Null present | Oosterhout | Chakraborty | Brookfield 1 | Brookfield 2 |  |
| quru-CA-2P24* | No | 0.02 | 0.02 | 0.01 | 0.01 |  |
| quru-CA-3A05* | No | -0.02 | -0.00 | -0.00 | 0 |  |
| FIR028 | Yes | 0.14 | 0.17 | 0.13 | 0.24 |  |
| quru-GA-1C06* | No | -0.01 | -0.00 | -0.00 | 0.04 |  |
| Qr_C0057 | Yes | 0.06 | 0.07 | 0.05 | 0.05 |  |
| Qr_C0332 | Yes | 0.07 | 0.08 | 0.0 | 0.05 |  |
| FS_C2660 | No | 0.04 | 0.03 | 0.02 | 0.02 |  |
| FS_C8183 | No | 0.00 | 0.00 | 0.00 | 0.00 |  |
| PIE223 | No | 0.02 | 0.02 | 0.02 | 0.04 |  |
| GOT040 | No | 0.01 | 0.01 | 0.01 | 0.04 |  |
| PIE125 | No | 0.00 | 0.006 | 0.00 | 0.00 |  |

*:gSSRs

| **Table S4.** Genetic variation values across all samples for each nSSR markers. | | | | | | | | | | | |
| --- | --- | --- | --- | --- | --- | --- | --- | --- | --- | --- | --- |
|  | quru-CA-2P24 | quru-CA-3A05 | FIR028 | quru-GA-1C06 | Qr_C0057 | Qr_C0332 | FS_C2660 | FS_C8183 | PIE223 | GOT040 | PIE125 |
| N_a_ | 2.00 | 6.33 | 16.67 | 6.33 | 6.00 | 3.33 | 4.33 | 2.33 | 7.67 | 5.67 | 2.00 |
| N_e_ | 1.44 | 2.62 | 9.47 | 2.71 | 3.31 | 1.91 | 1.90 | 1.12 | 3.55 | 2.05 | 1.44 |
| I | 0.48 | 1.25 | 2.45 | 1.20 | 1.36 | 0.71 | 0.81 | 0.22 | 1.44 | 0.88 | 0.48 |
| H_o_ | 0.29 | 0.61 | 0.64 | 0.64 | 0.61 | 0.40 | 0.43 | 0.11 | 0.69 | 0.49 | 0.29 |
| H_e_ | 0.30 | 0.62 | 0.89 | 0.63 | 0.69 | 0.48 | 0.46 | 0.11 | 0.72 | 0.51 | 0.30 |
| F_is_ | 0.03 | 0.01 | 0.28 | -0.02 | 0.12 | 0.16 | 0.06 | 0.00 | 0.04 | 0.04 | 0.07 |
| N_a_: number of Different Alleles; N_e_: Number of Effective Alleles; I, Shannon index; H_o_, observed heterozygosity; H_e_: expected heterozygosity; F_is_, Inbreeding Coefficient. | | | | | | | | | | | |

| **Supplementary Table S5.** Genetic variation at four cpDNA loci for each marker in the four sub-populations. | | | | | | | |
| --- | --- | --- | --- | --- | --- | --- | --- |
| Sub-populations | Locus | N | N_a_ | N_e_ | I | h | uh |
| Talesh–Mardab | QRcp028 | 42 | 4.00 | 1.27 | 0.47 | 0.21 | 0.22 |
|  | QRcp029 | 42 | 2.00 | 1.32 | 0.41 | 0.24 | 0.25 |
|  | ccmp6 | 42 | 1.00 | 1.00 | 0.00 | 0.00 | 0.00 |
|  | μcd4 | 42 | 3.00 | 1.27 | 0.44 | 0.21 | 0.22 |
|  | Mean | 42 | 2.50 | 1.22 | 0.33 | 0.17 | 0.17 |
| Lahijan–Nur | QRcp028 | 65 | 2.00 | 1.81 | 0.64 | 0.44 | 0.45 |
|  | QRcp029 | 65 | 2.00 | 1.81 | 0.64 | 0.44 | 0.45 |
|  | ccmp6 | 65 | 2.00 | 1.81 | 0.64 | 0.44 | 0.45 |
|  | μcd4 | 65 | 2.00 | 1.81 | 0.64 | 0.44 | 0.45 |
|  | Mean | 65 | 2.00 | 1.81 | 0.64 | 0.44 | 0.45 |
| Haraz–Naka | QRcp028 | 38 | 2.00 | 1.11 | 0.20 | 0.10 | 0.10 |
|  | QRcp029 | 38 | 2.00 | 1.05 | 0.12 | 0.51 | 0.05 |
|  | ccmp6 | 38 | 2.00 | 1.11 | 0.12 | 0.51 | 0.05 |
|  | μcd4 | 38 | 2.00 | 1.05 | 0.20 | 0.10 | 0.10 |
|  | Mean | 38 | 2.00 | 1.08 | 1.16 | 0.07 | 0.07 |
| Qarasu–Gorgan | QRcp028 | 84 | 1.00 | 1.00 | 0.00 | 0.00 | 0.00 |
|  | QRcp029 | 84 | 1.00 | 1.00 | 0.00 | 0.00 | 0.00 |
|  | ccmp6 | 84 | 1.00 | 1.00 | 0.00 | 0.00 | 0.00 |
|  | μcd4 | 84 | 1.00 | 1.00 | 0.00 | 0.00 | 0.00 |
|  | Mean | 84 | 1.00 | 1.00 | 0.39 | 0.23 | 0.24 |
| N, number of samples; N_a_, number of different alleles; N_e_, effective number of alleles; I, Shannon's Information Index; h, diversity; uh, Unbiased Diversity. | | | | | | | |
